# A Novel Mouse Model of *Campylobacter jejuni* Gastroenteritis Reveals Key Pro-inflammatory and Tissue Protective Roles for Toll-like Receptor Signaling during Infection

**DOI:** 10.1371/journal.ppat.1004264

**Published:** 2014-07-17

**Authors:** Martin Stahl, Jenna Ries, Jenny Vermeulen, Hong Yang, Ho Pan Sham, Shauna M. Crowley, Yuliya Badayeva, Stuart E. Turvey, Erin C. Gaynor, Xiaoxia Li, Bruce A. Vallance

**Affiliations:** 1 Division of Gastroenterology, British Columbia Children's Hospital, the Child and Family Research Institute and the University of British Columbia, Vancouver, British Columbia, Canada; 2 Department of Microbiology and Immunology, University of British Columbia, Vancouver, British Columbia, Canada; 3 Department of Pediatrics, British Columbia Children's Hospital and Child & Family Research Institute, University of British Columbia, Vancouver, British Columbia, Canada; 4 Department of Immunology, Cleveland Clinic Foundation, Cleveland, Ohio, United States of America; University of Michigan Medical School, United States of America

## Abstract

*Campylobacter jejuni* is a major source of foodborne illness in the developed world, and a common cause of clinical gastroenteritis. Exactly how *C. jejuni* colonizes its host's intestines and causes disease is poorly understood. Although it causes severe diarrhea and gastroenteritis in humans, *C. jejuni* typically dwells as a commensal microbe within the intestines of most animals, including birds, where its colonization is asymptomatic. Pretreatment of C57BL/6 mice with the antibiotic vancomycin facilitated intestinal *C. jejuni* colonization, albeit with minimal pathology. In contrast, vancomycin pretreatment of mice deficient in SIGIRR (*Sigirr^−/−^*), a negative regulator of MyD88-dependent signaling led to heavy and widespread *C. jejuni* colonization, accompanied by severe gastroenteritis involving strongly elevated transcription of Th1/Th17 cytokines. *C. jejuni* heavily colonized the cecal and colonic crypts of *Sigirr^−/−^* mice, adhering to, as well as invading intestinal epithelial cells. This infectivity was dependent on established *C. jejuni* pathogenicity factors, capsular polysaccharides (*kpsM*) and motility/flagella (*flaA*). We also explored the basis for the inflammatory response elicited by *C. jejuni* in *Sigirr^−/−^* mice, focusing on the roles played by Toll-like receptors (TLR) 2 and 4, as these innate receptors were strongly stimulated by *C. jejuni*. Despite heavy colonization, *Tlr4^−/−^/Sigirr^−/−^* mice were largely unresponsive to infection by *C. jejuni*, whereas *Tlr2^−/−^/Sigirr^−/−^* mice developed exaggerated inflammation and pathology. This indicates that TLR4 signaling underlies the majority of the enteritis seen in this model, whereas TLR2 signaling had a protective role, acting to promote mucosal integrity. Furthermore, we found that loss of the *C. jejuni* capsule led to increased TLR4 activation and exaggerated inflammation and gastroenteritis. Together, these results validate the use of *Sigirr^−/−^* mice as an exciting and relevant animal model for studying the pathogenesis and innate immune responses to *C. jejuni*.

## Author Summary

Research into the key virulence strategies of the bacterial pathogen *Campylobacter jejuni*, as well as the host immune responses that develop against this microbe have, in many ways, been limited by the lack of relevant animal models. Here we describe the use of *Sigirr* deficient (*^−/−^*) mice as a model for *C. jejuni* pathogenesis. Not only do *Sigirr^−/−^* mice develop significant intestinal inflammation in response to colonization by *C. jejuni*, but the ability of this pathogen to trigger gastroenteritis was dependent on key virulence factors. We also found that the induction of the inflammatory and Th1/Th17 immune responses to infection in these mice depended on specific Toll-like receptors, principally TLR4, which we identified as the main driver of inflammation. In contrast, TLR2 signaling was found to protect mucosal integrity, with *Tlr2^−/−^/Sigirr^−/−^* mice suffering exaggerated mucosal damage and inflammation. Notably, we found that *C. jejuni*'s capsule helped conceal it from the host's immune system as its loss led to significantly increased activation of host TLRs and exaggerated gastroenteritis. Our research shows that the increased sensitivity of *Sigirr* ^−/−^ mice can be used to generate a unique and exciting model that facilitates the study of *C. jejuni* pathogenesis as well as host immunity to this enteric pathogen.

## Introduction

*Campylobacter jejuni* is one of the leading bacterial causes of gastroenteritis in the world. Although responsible for the majority of food-borne bacterial infections in developed countries, and compared to many other common enteric bacterial pathogens, our understanding of the mechanisms underlying *C. jejuni*'s pathogenesis remains poorly defined [1]. One reason for our limited understanding is that *C. jejuni* appears to utilize unique pathogenic strategies, as it lacks many of the common toxins, effector proteins and virulence factors found in other pathogenic bacteria [1]. For example, cytolethal distending toxin is the only toxin so far identified within *Campylobacter* strains [1], [2] yet its potentially toxic role *in vivo* remains unclear [2]. Furthermore, a number of bacterial factors such as capsular polysaccharides [3], [4], lipo-oligosaccharides [4] and proteins such as CadF [5], Peb1 [6], [7], JlpA [8] and the *Campylobacter* invasive antigens (Cia) [1], [9]–[11], have all been studied *in vitro* for roles in *C. jejuni* cell adhesion and invasion, and in the existing commensal colonization models, however whether they play any role in pathogenicity *in vivo* is largely unknown.

Indeed, the study of *C. jejuni* pathogenicity has largely been limited by the lack of relevant and convenient animal models that can be used to replicate human disease [12]. While *C. jejuni* readily colonizes poultry, it does so in a commensal fashion, causing no disease and thus not providing significant insight into *C. jejuni* pathogenesis or how the host defends against these microbes. *Galleria mellonella* larvae, which are a common animal model used for the study of several bacterial pathogens have been applied to *C. jejuni* [13], [14], but their relevance in modeling vertebrate enteric infection is limited. While colostrum-deprived piglets [15], as well as ferrets [16] have been used to model *C. jejuni* infection with some success, their use is limited by the difficulty obtaining and maintaining these animals, and a lack of immunologic and genetic tools to aid in studying the host response to infection.

Mice would normally provide a preferred infection model system; however, they have repeatedly proven resistant to pathogenic infection by *C. jejuni*, and many strains are unable to even be reliably colonized [17]. The basis for their resistance to *C. jejuni* colonization appears to at least partially reflect active competition from the resident intestinal microbiota, thereby preventing *C. jejuni* from establishing a niche within the murine gut [17]–[19]. Secondly, the murine immune system has proven very tolerant to the presence of *C. jejuni* and in wild-type (WT) mice, their presence only rarely elicits any overt intestinal inflammation [20], [21]. To overcome this tolerance, several groups have tested genetically manipulated mice that develop exaggerated inflammatory responses to bacteria, such as IL-10-deficient (*Il-10^−/−^*) mice [20]. While *Il-10^−/−^* mice can be colonized by *C. jejuni*, resulting in severe enterocolitis, the loss of IL-10 dramatically alters the murine immune system. As a result, their immune system is unable to effectively clear *C. jejuni* from the GI tract, leading to chronic colonization rather than the acute infections seen in humans. Moreover the immune systems of *Il-10^−/−^* mice are so sensitive that the presence of any commensal microbe can potentially trigger spontaneous enterocolitis [21]. The oral gavage and intraperitoneal injections of MyD88-deficient mice, have also been employed for the study of *C. jejuni* colonization and dissemination in mice [22]–[24], but encounter the reverse limitation of *Il-10^−/−^* mice, where the immune response is attenuated, allowing for colonization of the intestine or systemic sites with limited host responses. This provides utility for the study of colonization, but not immunity and the development of inflammation in response to *C. jejuni*.

Recently we showed that mice deficient in Single IgG IL-1 Related Receptor (SIGIRR) exhibit increased susceptibility to infection by two natural enteric bacterial pathogens of mice, namely *Citrobacter rodentium* and *Salmonella enterica serovar* Typhimurium [25]. In both mice and humans, SIGIRR is highly expressed by intestinal epithelial cells and acts as a negative regulator of MyD88-dependent signaling, thus acting to dampen signaling by most Toll-like receptors as well as interleukin (IL)-1R [25]–[27]. In the absence of SIGIRR, when these receptors are activated, their downstream signaling is increased, resulting in increased innate inflammatory responses [26], [27]. In the context of *C. rodentium* and *S.* Typhimurium infections, we found that *Sigirr−/−* mice not only developed exaggerated forms of infectious colitis, but they were also infected much more rapidly and with much lower infectious doses than WT mice. This heightened susceptibility was shown to reflect exaggerated antimicrobial responses by *Sigirr^−/−^* mice that were surprisingly ineffective against pathogens, but instead depleted the competing commensal microbes [25], dramatically reducing the microbiota based resistance to intestinal pathogen colonization.

Based on their heightened susceptibility to natural bacterial pathogens of mice, we examined whether *Sigirr^−/−^* mice could potentially serve as an infection model for the human pathogen *C. jejuni*. Although orally delivered *C. jejuni* were able to sporadically colonize *Sigirr^−/−^* mice, antibiotic pretreatment was found to facilitate pathogen colonization, leading to acute gastroenteritis in infected *Sigirr^−/−^* mice. We confirmed that *C. jejuni* primarily activates the innate receptors TLR2 and TLR4 [28]–[31], and found that TLR4 signaling was responsible for most of the inflammatory changes seen during infection. In addition to the requirement for innate signaling, the development of gastroenteritis was also dependent on the activity and pathogenicity of *C. jejuni*. In infections with *C. jejuni* mutants deleted for *flaA* (flagella) [32] or *kpsM* (capsular polysaccharide) [31], [33], [34], the ability of *C. jejuni* to cause gastroenteritis was significantly altered. Together, these results validate the use of *Sigirr^−/−^* mice as an exciting and relevant animal model for studying innate immune responses to *C. jejuni*, as well as for the study of pathogenicity factors governing infection by this microbe.

## Results

### *Campylobacter jejuni* colonizes and infects the intestines of *Sigirr^−/−^* mice

**Figure 1:**
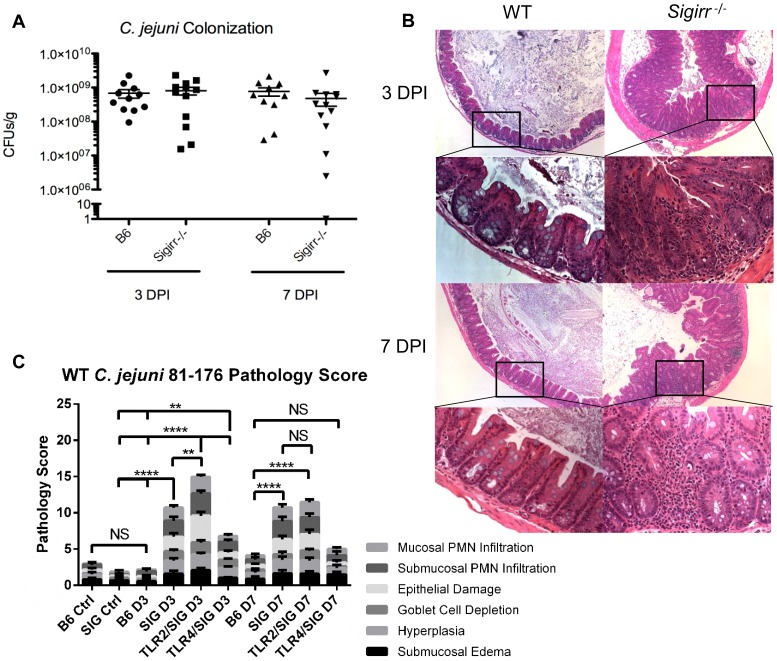
Colonization of WT and *Sigirr−/−* mice by *C. jejuni* 81–176, 3 and 7 DPI. (A) High numbers (∼10^9^ CFUs/g) of *C. jejuni* were recovered at both 3 and 7 DPI from the ceca of infected mice that were pre-treated with 5 mg of vancomycin. No statistically significant differences in numbers were found between WT and *Sigirr^−/−^* mice as indicated by a t-test, p>0.05. n = 10 or 11 WT mice, and 12 or 13 *Sigirr^−/−^* mice for 3 and 7 DPI respectively. (B) H&E stained, formalin-fixed histological sections of ceca recovered from WT or *Sigirr^−/−^* mice 3 and 7 DPI. Upper panels are ×100 magnification, while lower panels are ×400 magnification. (C) Pathological scoring was done by two blinded observers, using H&E stained, formalin-fixed cecal tissue sections. Each condition represents a minimum of three separate experimental replicates, with 2–3 mice per experiment for a total of 6–9 mice per group. Control mice were used as a reference and consisted of 3 uninfected mice, pre-treated with a single dose of 5 mg/100 µl vancomycin, and euthanized 3 days post-treatment. WT (B6) mice did not exhibit any significant signs of inflammation, while *Sigirr^−/−^* and *Tlr2^−/−^/Sigirr^−/−^* mice showed a significant increase relative to the uninfected *Sigirr^−/−^* control, both 3 and 7 DPI. *Tlr4^−/−^/Sigirr^−/−^* mice showed a statistically significant increase, relative to control mice at 3 DPI only, but even at 3 DPI, were significantly less than either *Sigirr^−/−^* and *Tlr2^−/−^/Sigirr^−/−^* mice. Statistical significance was determined using a two-way ANOVA and a Bonferroni post-test (NS p>0.05, *p<0.05, ** p<0.01, *** p<0.001, **** p<0.0001).

The murine intestine is thought to be highly resistant to oral infection by *C. jejuni*, based primarily on the ability of the resident gut microbiota to outcompete any incoming *C. jejuni* [17], [35]. Our experiments support this concept, as we found infrequent and inconsistent *C. jejuni* colonization of conventionally housed WT C57BL/6 mice following oral inoculation with our wild-type *C. jejuni* strain 81–176 (10^7^ CFU) (data not shown). To overcome this barrier to colonization, we pretreated WT C57BL/6 mice with vancomycin by oral gavage prior to inoculation with *C. jejuni*. Previous research by Russell et al. [36] showed that oral vancomycin treatment depleted *Bacteroidetes* and *Clostridia* from the intestines of mice while promoting the overgrowth of *Lactobacilli* [36]. Vancomycin pretreatment has also been shown to promote *S*. Typhimurium colonization and colitis in a fashion similar to streptomycin pretreatment [37]. Following oral inoculation with *C. jejuni*, we found the vancomycin pretreated WT mice exhibited consistent and robust pathogen colonization in their ceca (Figure 1a) and colons (Figure S1a). Despite their high levels of colonization, minimal signs of inflammation were observed (Figure 1b and c), consistent with previously published analyses of *C. jejuni*-colonized immunocompetent mice.

Considering that *Sigirr^−/−^* mice exhibit impaired colonization resistance against several murine enteric bacterial pathogens, we tested their susceptibility to *C. jejuni* infection/colonization. We again saw only sporadic colonization in some mice, but in contrast to WT mice, we also saw occasional signs of intestinal inflammation and other forms of pathology but the results were insufficiently reproducible to provide a reliable model (data not shown). We therefore tested the impact of pretreating *Sigirr−/−* mice with vancomycin, as previously described for WT mice. We noted that vancomycin induced a similar change in the intestinal microbiota of *Sigirr^−/−^* mice as we had found for WT mice (Figure S2). Four hours after vancomycin pretreatment, we orally infected *Sigirr^−/−^* mice along with WT mice with approximately 10^7^ CFU of *C. jejuni* 81–176. We euthanized the mice at 3 and 7 days post-infection, assessing pathogen burden in the cecum (Figure 1a), colon, ileum, mesenteric lymph nodes (MLN), spleen and feces (Figure S1a–e). Both WT and *Sigirr^−/−^* mice were quickly colonized, with both strains of mice reaching cecal colonization levels of approximately 10^9^ CFU/g within 3 days. In *Sigirr^−/−^* mice, colonization numbers usually peaked within 7–9 days, and began to drop significantly by 2–3 weeks post-infection, with low levels of *C. jejuni* (<10^4^ CFUs/g) found beyond 3 weeks (Figure S3a). WT mice maintained high and relatively unchanging pathogen burdens for at least 25 days (Figure S3a). Colonization in the colon was similar to the cecum, whereas relatively fewer *C. jejuni* were recovered from the ileum (Figure S1b). Fecal samples taken just prior to euthanization, and throughout the infection, proved to be largely representative of the colonization of both the cecum and colon, with the numbers more closely resembling the numbers recovered from the colon (Figure S1e and f). *C. jejuni* were also occasionally recovered in low numbers from the MLN and rarely from the spleen (Figure S1c, d), indicating that even with a high pathogen burden in the gut, *C jejuni* did not readily go systemic.

Despite carrying similar pathogen burdens, the macroscopic pathology resulting from *C. jejuni* colonization was dramatically more severe in the *Sigirr^−/−^* mice as compared to WT mice. Although neither mouse strain exhibited significant weight loss (>10%) (Figure S3b) or other severe signs of morbidity, the ceca and proximal colons of the *Sigirr* ^−/−^ mice were overtly inflamed and often devoid of stool contents. In mice at the height of infection, the stool itself often became noticeably softer and sticky. Significant enlargement of the mesenteric lymph nodes was also noted in infected *Sigirr* ^−/−^ mice (Figure S4). In comparison, control mice treated with vancomycin, but not receiving *C. jejuni*, exhibited no significant signs of intestinal pathology 3 or 7 days post antibiotic treatment (Figure 1c and data not shown). As expected, histology revealed few, if any, signs of cecal inflammation in WT mice at 3 DPI (Figure 1b), and only very mild signs of inflammation at 7 DPI, despite their heavy pathogen burden. In contrast, the cecal pathology and inflammation observed in infected *Sigirr* ^−/−^ mice was very severe at both 3 and 7 DPI, including submucosal edema, crypt hyperplasia and widespread immune/inflammatory cell infiltration (Figure 1c). In some cases, the *Sigirr* ^−/−^ mice developed focal cecal ulcers, accompanied by bleeding into the lumen (Figure 1b). The severe damage was focused within the cecum and proximal colon, with only minimal signs of inflammation appearing elsewhere in the intestine.

**Figure 2:**
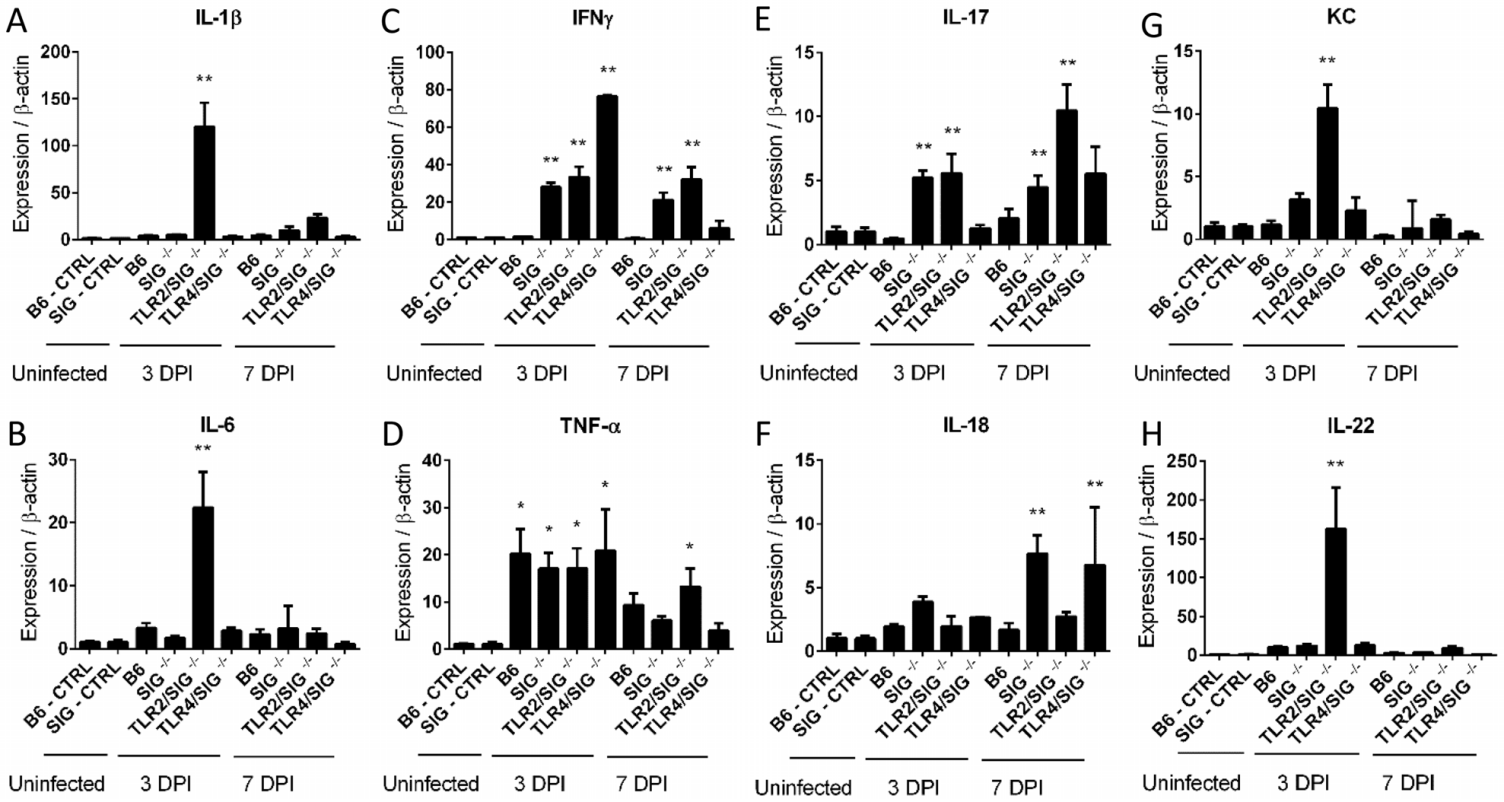
Cytokine production in infected mice. (A–H) RT-qPCR conducted on RNA extracted from the ceca of uninfected control or infected mice. Controls are the pooled results of 9, vancomycin pre-treated, but uninfected mice, euthanized 3 days post-treatment. All infected mice represent the average results of 3 independent experiments, each of which include the pooled RNA of 2–3 mice, for 6–9 mice total for each mouse strain, euthanized either 3 or 7 DPI. Statistical significance was determined using a One way ANOVA with a Bonferroni post-test. * p<0.05 relative to WT (B6) or *Sigirr^−/−^* uninfected control mice. ** p<0.05 relative to the infected WT (B6) mice euthanized on the same DPI in addition to the uninfected control mice.

When we measured gene transcript levels of several key cytokines, we found that despite the lack of overt inflammation in the infected WT mice, they still showed upregulated gene transcript levels for a number of cytokines compared to uninfected mice, most notably TNFα, indicating that the WT mice were not completely unresponsive to the presence of *C. jejuni*. However these responses were insufficient to trigger overt signs of inflammation (Figure 2). The infected *Sigirr* ^−/−^ mice also showed elevated cytokine gene transcripts at levels significantly higher than those seen in WT mice. Notably, at both 3 and 7 DPI, the *Sigirr* ^−/−^ mice showed significantly higher mRNA levels for IL-17A, TNF-α and Interferon gamma (IFNγ), indicative of a stronger inflammatory response. We also observed higher transcription of the neutrophil chemoattractant KC, as well as the cytokines IL-1β, IL-18 and IL-22, though the variability between mice prevented the demonstration of statistical significance.

### *C. jejuni* localizes within the cecal crypts of *Sigirr* ^−/−^ mice

**Figure 3:**
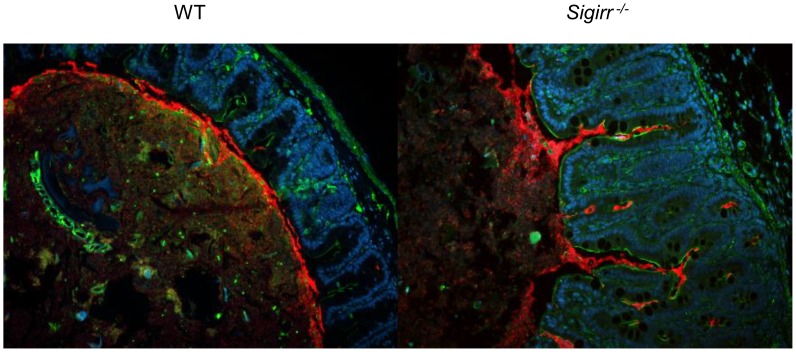
Immunofluorescence staining of *C. jejuni* cecal colonization *in vivo*. Formalin-fixed tissue sections of ceca obtained from *C. jejuni*-infected WT and *Sigirr^−/−^* mice 7 DPI at ×200 magnification. Cell nuclei are stained with DAPI (blue), epithelial cells are outlined with antibodies specific to β-actin (green), and *C. jejuni* (red) are clearly visible around the edge of the lumen and into the cecal crypts.

To better define the cause of the exaggerated tissue pathology suffered by infected *Sigirr* ^−/−^ mice, we explored the localization of the colonizing *C. jejuni* and whether it differed with that in WT mice. Staining for *C. jejuni* in intestinal tissue sections of WT mice at both 3 and 7 DPI revealed the bacteria were largely limited to the intestinal lumen, with rare *C. jejuni* found in only 26.5% (70/264) of crypts. We also noted *C. jejuni* accumulating in the mucus layer, whereas relatively few microbes were found in direct contact with the intestinal epithelium or penetrating the cecal or colonic crypts (Figure 3). Conversely, in the *Sigirr* ^−/−^ mice, *C. jejuni* were not only found within the intestinal lumen and the mucus layer, but large numbers were also seen penetrating deep within cecal and colonic crypts (Figure 3). In these mice, 56.2% (82/146) of crypts were found to be heavily colonized by *C. jejuni*.

### *C. jejuni* adhere to as well as invade the intestinal epithelium of *Sigirr* ^−/−^ mice

**Figure 4:**
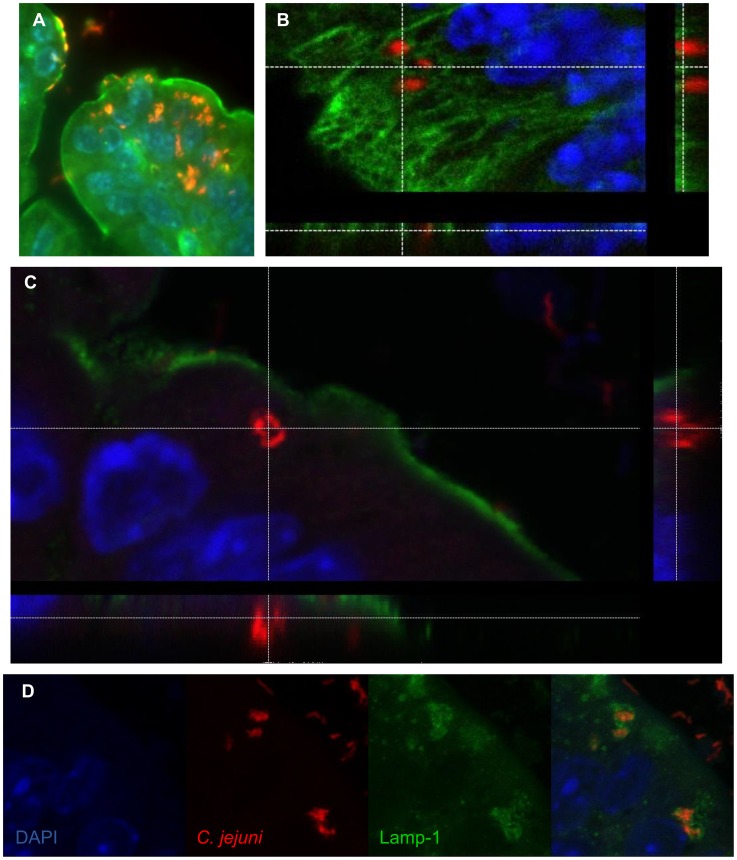
Immunofluorescent staining of intracellular *C. jejuni in vivo*. (A) Intracellular *C. jejuni* are visible in the cecal epithelium. *C. jejuni* (red), are visible against the β-actin (green) and the nuclei (DAPI, blue) of the cecal epithelium of a *Sigirr^−/−^* mouse, ×1000 magnification. (B) Confocal image of *C. jejuni* (red) present within epithelial cells of the colon of a *Sigirr^−/−^* mouse, highlighted against the Cytokeratin 19 of the cytoskeleton (green) and the nuclei (blue), with the z-stack cross-section indicating the *C. jejuni* within the cell. (C) Cross-section of a Z-stack, of a colonic epithelial cell of a *Sigirr^−/−^* mouse. The internalized *C. jejuni* (red) are clearly visible within the cytoplasm of the cell, as outlined by the β-actin (green) along the edge of the cell. (D) Internalized *C. jejuni* (red) co-localize with LAMP-1 positive (green) vesicles present within epithelial cells of a *Sigirr^−/−^* mouse colon.

When we examined the localization of *C. jejuni* within *Sigirr* ^−/−^ tissues more closely, we observed that large numbers of the bacteria were in direct contact with the intestinal epithelium, particularly within crypts (Figure 3a). By co-staining for *C. jejuni* antigens along with either β-actin or cytokeratin 19, we could clearly visualize the cytoskeleton of the epithelial cells, relative to the localization of the *C. jejuni*. In addition to adherent *C. jejuni*, we also visualized *C. jejuni* co-localizing with and potentially within the epithelial layer (Figure 4a). To address whether these *C. jejuni* were intracellular, we examined the stained cells using confocal microscopy, to determine whether they were in fact internalized (Figure 4b and c). Indeed, in the X, Y and Z axes, labeled *C. jejuni* were present inside epithelial cells, often organized into spherical foci, suggesting their localization within a vesicle or phagosome (Figure 4b and c). Previous studies have identified Lamp-1, a lysosome-associated membrane protein as a marker for intracellular *S*. Typhimurium containing vacuoles [38], [39] as well as for phagosomes containing *C. jejuni* [40], [41] inside cultured epithelial cells. To address whether a similar structure was present *in vivo*, we stained for Lamp-1 [42], and clearly observed internalized *C. jejuni* within epithelial cells to be surrounded by Lamp-1 positive membrane structures (Figure 4d). Although the precise numbers of internalized *C. jejuni* present in a tissue section varied, we observed intracellular *C. jejuni* in all infected *Sigirr* ^−/−^ mice tested.

### Campylobacter virulence factors are necessary for successful infection

While our data showed that SIGIRR deficiency facilitated the ability of *C. jejuni* to adhere to and infect intestinal epithelial cells *in vivo*, resulting in overt gastroenteritis, it was unclear whether the resulting pathology depended on *C. jejuni* pathogenicity factors. To test this, we inoculated our WT and *Sigirr* ^−/−^ mice with two previously well-characterized *C. jejuni* mutants: *ΔkpsM* and *ΔflaA*, as well as the complemented strains for each mutant. The *kpsM* gene encodes the permease of the capsule polysaccharide ABC transporter. This gene deletion results in the loss of the entire capsule surrounding the microbe, which is thought to be a key virulence-associated cellular structure [33], [43]. The *ΔflaA* flagellar mutant lacks the primary flagellin protein, and although expression of the secondary FlaB flagellin continues, the result is a truncated flagellum and a significant loss of motility [44]. This phenotype has been previously associated with an inability to invade epithelial cells *in vitro* [32], and defective colonization of chicks [45].

**Figure 5:**
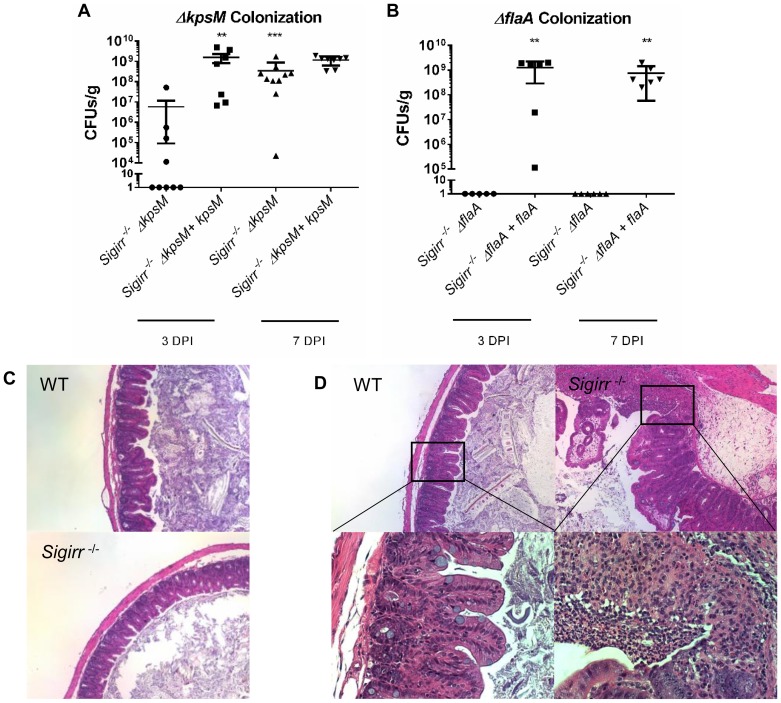
Colonization of WT and *Sigirr−/−* mice by *ΔkpsM* and *ΔflaA*. Colonization of WT and *Sigirr^−/−^* mice by *ΔkpsM* (A) and *ΔflaA* (B) and their respective complemented strains (*ΔkpsM*+*kpsM* and *ΔflaA*+*flaA*), at both 3 and 7 DPI. The *ΔkpsM* mutant exhibited reduced colonization at 3 DPI only, while the *ΔflaA* mutant was unable to colonize at either 3 or 7 DPI. The complemented *ΔflaA+flaA* colonized at high numbers, similar to wild-type. Statistical significance was determined by a Mann-Whitney test, ***p<0.001. n = 7–10 mice for the *ΔkpsM* mutant and complement, n = 5–7 mice for the *ΔflaA* mutant and complement. (C) H&E stained histological sections of ceca recovered from WT or *Sigirr^−/−^* mice infected with *C. jejuni ΔflaA* at ×100 magnification. No noticeable inflammation was evident in either WT or *Sigirr^−/−^* mice infected with *C. jejuni ΔflaA*. (D) H&E stained histological sections of ceca recovered from WT or *Sigirr^−/−^* mice infected with *C. jejuni ΔkpsM* 7 DPI. Upper panels are ×100 magnification, while lower panels are ×400 magnification. WT mice did not exhibit signs of inflammation when infected with *C. jejuni ΔkpsM*, however *Sigirr^−/−^* mice exhibited signs of severe inflammation at 7 DPI.

We initially observed significant shifts in colonization for each of the mutant strains tested. Whereas wild-type *C. jejuni* readily colonized the intestines of *Sigirr* ^−/−^ mice, each of the mutant strains suffered colonization defects (Figure 5a and b, Figure S5a). The *ΔkpsM* mutant was significantly impaired at 3 DPI, but approached WT numbers by 7 DPI. The complemented version of this mutant was significantly less impaired for colonization at 3 DPI, and more closely resembled the colonization potential of the wild-type strain. Conversely, the *ΔflaA* flagellar mutant was severely impaired in colonization and was completely lost from the intestine by 3 DPI and remained absent at 7 DPI. The mutant and complemented strains were also assessed for growth *in vitro*, and neither mutant exhibited growth defects (Figure S5b).

In terms of pathology, each mutant exerted a substantially different effect on the gastroenteritis seen in infected *Sigirr* ^−/−^ mice. As might be expected given the severe colonization defect, the *ΔflaA* mutant did not elicit any significant inflammation or pathology (Figure 5c). In contrast, despite the *ΔkpsM* mutant suffering delayed colonization, it still caused overt gastroenteritis at 7 DPI that was in fact significantly worse than that seen following infection with wild-type *C. jejuni* (Figure 5d). To explore the basis for this exaggerated pathology, we next examined how the immune system is stimulated during *in vivo C. jejuni* infection.

### TLR2 and TLR4 are key players in the inflammatory response to *C. jejuni*

**Figure 6:**
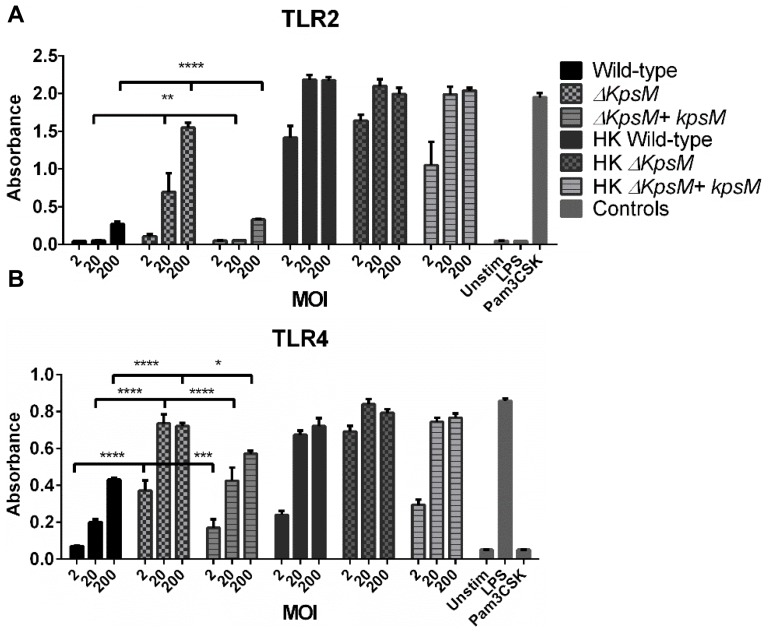
TLR2 and 4 reporter assays. HEK-Blue hTLR2 (A) and HEK-Blue hTLR4 (B) reporter cell lines were exposed for 4 hrs to either live and heat-killed wildtype *C. jejuni* 81–176, *ΔkpsM* or *ΔkpsM+kpsM*. The wild-type *C. jejuni* stimulates both TLR2 and TLR4 in a dose-dependent fashion. The *ΔkpsM* mutant significantly increased the signaling by both TLR2 and TLR4, as indicated by the assay, with the increase in stimulation also being in a dose-dependent manner, except for the TLR4 assay where the readers were near the maximum for both the 20 and 200 MOI readings. The complemented *ΔkpsM+kpsM* strain completely restored the wild-type phenotype with TLR2 and mostly restored the phenotype with TLR4. Values represent the mean of three independent experiments and statistical significance was determined by a two-way ANOVA with a Bonferroni post-test. (* p<0.05, ** p<0.01, *** p<0.001, **** p<0.0001).

**Figure 7:**
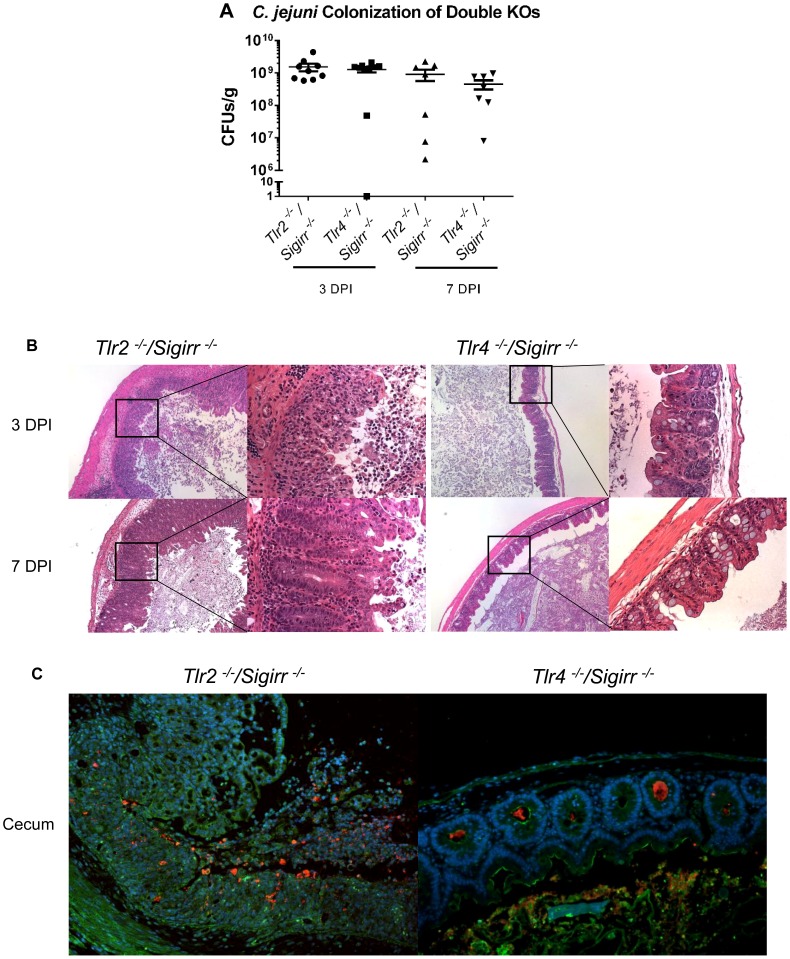
Pathology of *Tlr2^−/−^/Sigirr^−/−^* and *Tlr4^−/−^/Sigirr^−/−^* mice infected with *C. jejuni*. (A) High numbers (∼10^9^ CFUs/g) of *C. jejuni* were recovered from the ceca of infected *Tlr2^−/−^/Sigirr^−/−^* and *Tlr4^−/−^/Sigirr^−/−^* mice that were pre-treated with 5 mg/ml of vancomycin. No statistically significant differences in numbers were found between WT, *Sigirr^−/−^, Tlr2^−/−^/Sigirr^−/−^* and *Tlr4^−/−^/Sigirr^−/−^* mice as indicated by t-test, p>0.05. n = 7–9 mice per condition. (B) H&E stained histological sections of ceca recovered from *Tlr2^−/−^/Sigirr^−/−^* and *Tlr4^−/−^/Sigirr^−/−^* mice 3 or 7 DPI. Panels on the left are ×100 magnification, while the right panels are ×400 magnification. Very severe inflammation is apparent in the *Tlr2^−/−^/Sigirr^−/−^* mice both 3 and 7 DPI. Few signs of inflammation are evident in the *Tlr4^−/−^/Sigirr^−/−^* mice 3 DPI, and no signs of inflammation are apparent 7 DPI. (C) Immunofluorescence staining of formalin-fixed tissue sections of ceca obtained from *C. jejuni*-infected *Tlr2^−/−^/Sigirr^−/−^* and *Tlr4^−/−^/Sigirr^−/−^* mice 3 DPI at ×200 magnification. Sections are stained for DAPI (blue), β-actin (green), and *C. jejuni* (red). *C. jejuni* is clearly visible in the crypts of the *Tlr4^−/−^/Sigirr^−/−^* mice, but with few signs of inflammation. The *Tlr2^−/−^/Sigirr^−/−^* mice lose much of their crypt structure, due to epithelial cell loss and immune cell infiltration, however, *C. jejuni* is still clearly visible intermixed with immune cells in what remains of the cecal crypts.

Previous research has shown that *C. jejuni* activates several Toll-like receptors (TLR) including TLR2 and TLR4, and that TLR activation may play a key role in regulating host inflammatory responses to *C. jejuni* [21], [28]–[31]. To confirm that our wild-type *C. jejuni* strain (81–176) stimulated these TLRs, we used HEK-TLR2 and HEK-TLR4 reporter cells with a NF-κB/AP-1 inducible reporter- SEAP to measure stimulation of TLR2 and TLR4 *in vitro*. We observed significant stimulation of both receptors by *C. jejuni* 81–176, consistent with previously published results by Maue et al. [31] (Figure 6). To explore the impact that this activation might play in our infection model, we infected *Tlr2^−/−^/Sigirr^−/−^* and *Tlr4^−/−^/Sigirr^−/−^* mice. Although the *Tlr4^−/−^/Sigirr^−/−^* mice were heavily colonized by *C. jejuni*, (Figure 7a) they proved largely unresponsive to the pathogen, exhibiting few if any signs of the gastroenteritis seen in infected *Sigirr* ^−/−^ mice (Figure 7b). Notably, these mice showed little response to infection even at the gene transcriptional level. While these mice did exhibit significantly elevated expression of TNFα and IFNγ at 3 DPI, by 7 DPI, their expression of these, and other pro-inflammatory cytokines had decreased to levels similar to those in uninfected controls (Figure 2).

Conversely, the *Tlr2^−/−^/Sigirr^−/−^* mice were significantly more sensitive to *C. jejuni* infection, even compared to infected *Sigirr^−/−^* mice (Figure 7b and c), suffering exaggerated gastroenteritis by 3 DPI that involved worsened edema, crypt hyperplasia and inflammatory cell infiltration, including large numbers of neutrophils. Moreover, there were frequent signs of ulceration in these mice, along with loss of crypt structure and overall loss of epithelial integrity. Pathological scoring of tissues confirmed that the damage suffered by the *Tlr2^−/−^/Sigirr^−/−^* mice was significantly more severe than that seen in WT mice, and even more than that of *Sigirr^−/−^* mice at 3 DPI, though the severity of their inflammation was reduced by 7 DPI, leaving it similar in severity to that seen in *Sigirr^−/−^* mice at this time point. Consistent with their severe pathology, we observed a dramatic induction of inflammatory cytokine genes within the ceca of *Tlr2^−/−^/Sigirr^−/−^* mice at 3 DPI (Figure 2). This included significantly elevated levels of IL-1β, IL-6, IFNγ, KC, IL-22, IL17 and TNF-α gene transcripts, particularly at 3 DPI, although expression of many of these cytokines dropped by 7 DPI (Figure 2). Interestingly, the localization of *C. jejuni* was similar amongst all three SIGIRR-deficient mouse strains, with the *C. jejuni* seen in large numbers deep within cecal and colonic crypts, as well as inside intestinal epithelial cells (Figure 7c and data not shown). *C. jejuni* colonization of crypts in the *Tlr2^−/−^/Sigirr^−/−^* and *Tlr4^−/−^/Sigirr^−/−^* strains was comparable to the *Sigirr^−/−^* mice, with 41.7% (50/120) and 47.1% (66/140) of observed crypts being positively colonized, often with high numbers of bacteria in each crypt (Figure 7c). This indicates that the significant differences in pathology amongst the three SIGIRR deficient mouse strains were governed by the stimulation of the TLRs instead of by changes in the localization of the bacteria. We also assessed *Tlr2^−/−^* and *Tlr4^−/−^* single mutants for colonization and inflammation. Once again, pathogen burden was not affected by the mouse strain so long as it was accompanied by vancomycin pretreatment (data not shown). Unsurprisingly, *Tlr4^−/−^* were completely unresponsive to the presence of *C. jejuni*, exhibiting no inflammation (Figure S6). *Tlr2^−/−^* were much less responsive than *Tlr2^−/−^/Sigirr^−/−^* mice, but did show modest signs of inflammation by 7 DPI (Figure S6). Together, these results demonstrate that the majority of the inflammation seen in this model is driven by TLR4, whereas TLR2 signaling appears to play a protective role.

### TLR2 and TLR4 are activated to a higher degree by mutant *ΔkpsM C. jejuni*

**Figure 8:**
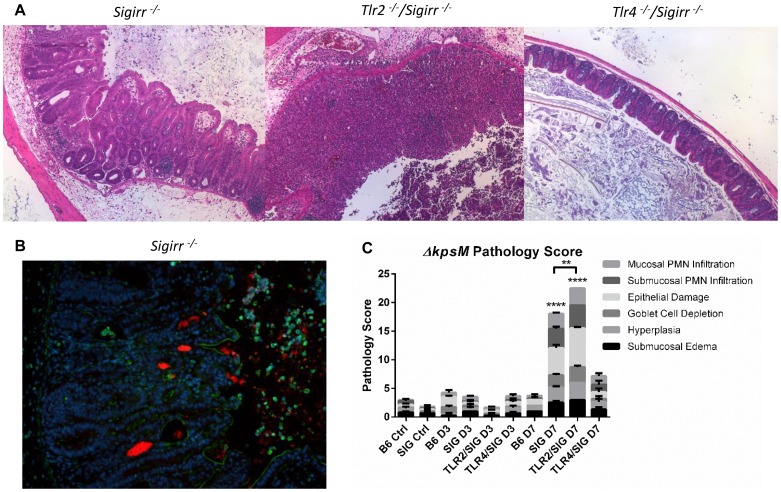
Colonization and pathology of *Sigirr^−/−^* and TLR-deficient mice by *C. jejuni ΔkpsM in vivo*. (A) H&E stained histological sections of ceca recovered from *Sigirr^−/−^*, *Tlr2^−/−^/Sigirr^−/−^* and *Tlr4^−/−^/Sigirr^−/−^* mice, colonized with *C. jejuni ΔkpsM* 7 DPI, at 100× magnification. Very severe inflammation is evident in the *Sigirr^−/−^* and *Tlr2^−/−^/Sigirr^−/−^* mice, however once again, no significant pathology was evident in the *Tlr4^−/−^/Sigirr^−/−^* mice. (B) Immunofluorescence of *Sigirr^−/−^* mice infected by *C. jejuni ΔkpsM*, 7 DPI. Sections are stained for DAPI (blue), β-actin (green), and *C. jejuni* (red). *Sigirr^−/−^* mice exhibit significant neutrophil infiltration, hyperplasia, and *C. jejuni ΔkpsM* is clearly visible in large masses within the cecal crypts. (C) Pathological scoring was done by two blinded observers, using H&E stained, formalin-fixed cecal tissue sections. Each condition represents a minimum of three separate experimental replicates, with 2–3 mice per experiment. Control mice were used as a reference and consisted of 3 uninfected mice, pre-treated with a single dose of 5 mg/100 µl vancomycin, and euthanized 3 days post-treatment. Only *Sigirr^−/−^* and *Tlr2^−/−^/Sigirr^−/−^* mice at 7 DPI showed a significant increase in pathology (****p<0.0001), relative to the uninfected *Sigirr^−/−^* control. The *Tlr2^−/−^/Sigirr^−/−^* mice also exhibited statistically significantly higher inflammation at 7 DPI relative to *Sigirr^−/−^* mice also at 7 DPI (**p<0.001). In contrast, none of the mouse strains at 3 DPI showed any statistically significant increase in pathology, relative to control mice. Statistical significance was determined using a two-way ANOVA and a Bonferroni post-test.

Based on previous data published by Rose et al. [34] and Maue et al. [31] we expected the capsule to play a role in modulating TLR responses to *C. jejuni*. To further explore the impact of TLR signaling during *C. jejuni* infection, we tested the effect of the *ΔkpsM* mutant on our TLR2 and TLR4 reporter cell lines. The *ΔkpsM* mutant stimulated both TLR2 and TLR4 to a significantly higher degree than the wild-type 81–176 strain with the complemented *ΔkpsM+kpsM* strain completely or nearly completely rescuing the mutant phenotype (Figure 6). To test whether these results translated to increased inflammation *in vivo*, we infected our different mouse strains with this mutant. As shown in Figure 8a, the *ΔkpsM* mutant elicited a very significant inflammatory response in the *Sigirr^−/−^* mice by 7 DPI. Moreover, it also caused exaggerated inflammation and pathology in *Tlr2^−/−^/Sigirr^−/−^* mice as compared to the effects of wild-type *C. jejuni*, yet once again there was little response in the *Tlr4^−/−^/Sigirr^−/−^* mice (Figure 8a). The localization of the *ΔkpsM* mutant *in vivo* was similar to that of wild-type *C. jejuni*, as it was frequently found in direct contact with the epithelium and deep within crypts (Figure 8b). These results were confirmed when cytokine transcript levels were assessed (Figure S7) Together, these data support previously published *in vitro* results [31], [34], and for the first time demonstrates that the *C. jejuni* capsule limits the host innate responses to this pathogen during the course of infection.

## Discussion

A lack of animal models, and in particular mouse models that replicate the gastroenteritis caused by *C. jejuni* infection in humans, has long been an impediment to the study of *C. jejuni* pathogenesis. Moreover, improved preclinical models of *C. jejuni* infection are a necessity to better define those host factors that protect against this pathogen. Here we demonstrate that the antibiotic vancomycin facilitates *C. jejuni*'s colonization of the mouse intestine, presumably through the removal of commensal microbes that promote resistance against *C. jejuni* colonization. Moreover, our studies validate the use of vancomycin pretreated *Sigirr^−/−^* mice as a model for *C. jejuni* infection and pathogenesis, as these mice develop acute gastroenteritis following infection. We were able to define the role of innate signaling in this model through the testing of *Tlr2^−/−^/Sigirr^−/−^* and *Tlr4^−/−^/Sigirr^−/−^* mice as well as clarify specific aspects of *C. jejuni* pathogenesis through the testing of mutant strains. Taken together, we demonstrate that by modulating the gut microbiota as well as the innate sensitivity of the murine intestine, we have been able to develop a reliable and exciting new animal model of *C. jejuni* infection.

WT mice, including the C57BL/6 strain used in this study, have in the past proven resistant to infection by *C. jejuni*, limiting their utility as an infection model [17], [35]. This limitation in the ability to colonize mice has been linked to the intestinal microbiota, and its ability to out-compete invading *C. jejuni*. Previous studies have explored how to overcome this barrier by using mice with a “humanized” microbiota [17], as well as germ-free mice and mice carrying a limited microflora [19], [21], [46]. In the current study, we used an approach successfully employed with other bacterial pathogens [37], using a single pre-treatment of the antibiotic vancomycin to disturb the murine microbiota sufficiently to allow *C. jejuni* to establish in the intestine. This colonization was, however, insufficient to produce an effective model of inflammation, as the colonized mice remained highly tolerant to the presence of *C. jejuni*, displaying few if any signs of inflammation, even in the presence of a high pathogen load.

Our previous studies identified *Sigirr^−/−^* mice as displaying increased susceptibility to infection by the natural mouse pathogens *S*. Typhimurium and *C. rodentium* in terms of both the severity of disease as well as pathogen burden [25]. Normally highly expressed by the intestinal epithelium, SIGIRR acts to dampen signaling through MyD88-dependent receptors such as most TLRs as well as IL-1R [26]. Thus SIGIRR expression is thought to help maintain the relative innate hypo-responsiveness of the intestinal epithelium. While the absence of SIGIRR does not lead to spontaneous intestinal inflammation, it does leave the epithelium more sensitive to microbial stimulation through TLRs. Previous studies identified TLR2 and TLR4 as being stimulated by *C. jejuni in vitro* [31], [34] and our study confirmed these two TLRs actively respond to *C. jejuni*. Moreover, TLR4 has been identified as being a major driver of inflammation in *C. jejuni*-infected *IL-10^−/−^* mice [21] and the present study found the gastroenteritis seen in infected *Sigirr^−/−^* mice is almost completely TLR4 dependent. In contrast, TLR2 signaling was found to protect the intestine from exaggerated injury, potentially by promoting responses in the epithelium that limit the damage caused by the TLR4 driven inflammation.

Taken together, there are several advantages to the use of the *Sigirr^−/−^* mouse over other models of *C. jejuni* infection. While *C. jejuni* readily colonizes the intestines of newborn chickens, it only does so in a commensal fashion, thus providing little insight into its pathogenesis or how it triggers gastroenteritis. While neonatal piglet and ferret models have been used successfully as models for infection [15], [16], both have significant limitations as these animals are difficult to acquire as well as handle, and there are few immunological or genetic tools available for these species. To circumvent these issues, mice remain one of the preferred animal species for use in research, but their resistance to *C. jejuni* colonization and disease has limited their utility in the field. The most successful mouse model of *C. jejuni* infection to date has been the *Il-10^−/−^* mouse, which has several features in its favor, including a very strong and reproducible inflammatory response [20], [21], [46]. However, it also suffers from several complications, notably the propensity of the *Il-10^−/−^* mice to develop spontaneous colitis as a reaction against their own microbiota [47], forcing researchers to use more inconvenient and costly germ-free conditions [21], [46]. Additionally, IL-10 has been shown to be a key cytokine in the resolution for inflammation following infection, meaning that *C. jejuni* infection in *IL-10^−/−^* mice is ultimately chronic and lethal to the mice, as opposed to the acute, self-limiting infection observed in humans.

Our use of *Sigirr^−/−^* mice addresses most of these issues. When orally inoculated with a relatively low dose of *C. jejuni*, the vancomycin pre-treatment allowed for reliable colonization, while only causing a temporary disruption in the microbiota. Although our previous research has identified a higher inflammatory “tone” in the *Sigirr^−/−^* mice, characterized by slightly higher expression of several pro-inflammatory cytokines [25], the *Sigirr^−/−^* mice themselves do not develop spontaneous colitis in response to their own microbiota as often occurs in *IL-10^−/−^* mice. When infected with *C. jejuni* they developed only an acute gastroenteritis, in keeping with the clinical effects of *C. jejuni* infection. Moreover, the gastroenteritis bears several hallmarks of *C. jejuni* infection, including prominent neutrophil infiltration into the infected tissues and lumen. Our assessment of *Tlr4^−/−^/Sigirr^−/−^* mice determined that the vast majority of the inflammation seen in this model is TLR4 dependent, which is in agreement with previous observations in gnotobiotic *IL-10* ^−/−^ mice [21].

In contrast to the modest responses seen in infected *Tlr4^−/−^/Sigirr^−/−^* mice, when we infected *Tlr2^−/−^/Sigirr^−/−^* mice, we observed a significantly exaggerated and accelerated form of gastroenteritis, especially at the early stages of infection (3 DPI). Correspondingly, these mice suffered increased pathology, including widespread loss of epithelial integrity, loss of crypt structure and frequent ulceration. This was accompanied by substantially increased pro-inflammatory cytokine expression. The most severe pathology was apparent at 3 DPI, with both cytokine expression and pathology dropping substantially by 7 DPI to the point where it was no longer significantly more severe than that seen in *Sigirr^−/−^* mice. These findings are intriguing as they suggest that TLR2 plays a protective role, at least during the early stages of *C. jejuni* infection. Previous studies have identified roles for TLR2 in the maintenance of epithelial tight junctions in the intestine, for example by increasing the production of Trefoil Factor 3 [48], along with other barrier protective proteins [49], [50]. As recently described by our laboratory, innate inflammatory responses in the GI tract appear to reflect a tenuous balance between damaging inflammatory signals and concurrent protective or tolerance inducing innate responses that limit the resulting tissue damage. It appears this is also the case during *C. jejuni* infection, with TLR2 playing a key role in limiting damage suffered by the host as its immune system tries to clear *C. jejuni* from the intestine [51].

Aside from exploring the host response to infection, an optimal *C. jejuni* infection model must be able to distinguish subtle aspects of *C. jejuni* pathogenicity. To address this issue, we infected our *Sigirr^−/−^* mice with *C. jejuni* strains lacking the ability to form a capsule, as well as a flagellar mutant. Regarding the *ΔflaA* flagellar mutant, previous studies have found *C. jejuni* mutants that are non-motile or suffer reduced motility are unable to effectively colonize the intestines of chicks [45], piglets [15], or wildtype mice [52], [53]. We therefore expected the *ΔflaA* strain to be impaired in colonization, and indeed, the *ΔflaA* mutant was unable to colonize the *Sigirr* ^−/−^ mice or cause any level of gastroenteritis. Precisely why this mutant was unable to colonize is an interesting question. We predominantly observe *C. jejuni* colonization in the mucus layer and into the crypts. Both leaving the lumen of the intestine and migration through the mucus layer would presumably require fully motile bacteria. It would appear that the inability of *C. jejuni* with reduced motility to reach and move through these niches results in a loss of colonization potential, even in mice with reduced microbiota competition.

In the case of the *ΔkpsM* mutant, it exhibited a delay in colonization as assessed at 3 DPI, but by 7 DPI, its pathogen load had increased to levels similar to WT *C. jejuni*. This could indicate a greater sensitivity of this mutant to the innate defenses in the gut. Previous work has already linked the loss of capsule to increased sensitivity to environmental factors such as osmotic stress [54], as well as antimicrobial factors [55]. However, the reduced colonization of *ΔkpsM* was transient, as the mutant quickly recovered to WT levels. Despite this initial delay, the *ΔkpsM* strain was able to elicit significantly more severe gastroenteritis than that seen with the wild-type *C. jejuni*. When we tested the *ΔkpsM* mutant in our *in vitro* reporter system, we found it stimulated both TLR2 and TLR4 to a significantly higher degree than that seen with WT *C. jejuni*. This result is consistent with previous findings [31], [34], suggesting a role for the capsule in reducing the exposure of pathogenic bacteria to the host's immune system by masking some of the TLR activating PAMPs. For example, Rose *et al*. determined that the presence of a capsule reduces cytokine expression by dendritic cells exposed to *C. jejuni in vitro* [34], and a similar observation was made by Maue *et al*. in an epithelial reporter cell line [31]. Here, for the first time *in vivo*, we demonstrate that the capsule does help conceal *C. jejuni* from the host's immune system, potentially as a means to limit host driven defenses as well as perhaps limit collateral tissue damage to the host.

One of the most intriguing findings from our study involved the localization of *C. jejuni* in the intestines of the *Sigirr^−/−^* mice. In WT mice, *C. jejuni* were found predominantly in the lumen, along with a clustering at the luminal surface of the mucus layer. Notably, relatively few *C. jejuni* were found in direct contact with the cecal or colonic epithelium, and few were seen penetrating the crypts. In contrast, in the *Sigirr* ^−/−^ mice we found large numbers of *C. jejuni*, not only within this mucus layer, but also penetrating and accumulating in large numbers at the base of the intestinal crypts. Similar observations were made in infected *Tlr2^−/−^/Sigirr^−/−^* mice, although in these mice, the exaggerated damage they suffered often left *C. jejuni* mixed within sloughed epithelial cells as well as phagocytosed inside neutrophils at sites of ulceration. This phenotype of crypt colonization was not restricted to mice developing severe inflammation, since *Tlr4^−/−^/Sigirr^−/−^* mice also displayed large numbers of bacteria within their cecal crypts. Thus the factor within *Sigirr^−/−^* mice that permits *C. jejuni* to colonize the crypts is not a result of overt inflammation, in contrast to recent studies of *S.* Typhimurium colonization of the murine intestine [56], but may be due instead to more subtle differences in the microenvironment of the crypts.

Aside from penetrating intestinal crypts, we also determined that *C. jejuni* could invade the intestinal epithelial cells of our *Sigirr* ^−/−^ mice. Intracellular *C. jejuni* were observed in both the cecum and colon of infected mice, and were usually seen in the more mature epithelial cells, at the top of crypts rather than at their base. Co-staining with β-actin or cytokeratin 19 confirmed that the *C. jejuni* were inside epithelial cells, while staining for LAMP-1 localized the internalized bacteria within LAMP-1 positive vesicles. Interestingly, the presence of intracellular *C. jejuni* did not on its own drive significant inflammation as internalized bacteria were also found in infected *Tlr4^−/−^/Sigirr^−/−^* mice, which exhibited no significant signs of inflammation. This indicates that intracellular *C. jejuni* can exist without causing overt inflammation or pathology; however, it remains possible that this cellular invasion can play a triggering role for the overt inflammation seen in infected *Sigirr^−/−^* and *Tlr2^−/−^/Sigirr^−/−^* mice.

Together, these studies provide new insight into the pathogenicity of *C. jejuni*, and how colonization by this microbe triggers an inflammatory reaction by its host. In conventional WT mice, the commensal microbiota provide colonization resistance against *C. jejuni* by outcompeting the invading pathogen. Through vancomycin treatment, we were able to readily disrupt this protection, but the WT mice remained substantially tolerant to the presence of *C. jejuni*, resulting in almost no inflammatory response. In contrast, in the absence of SIGIRR, the murine immune system proved dramatically more responsive to *C. jejuni*, potentially by increasing the sensitivity of epithelial expressed TLRs. Overall, the degree of inflammation that developed in the infected intestines of the *Sigirr^−/−^* mice appeared to correlate with the invasion by *C. jejuni* of the intestinal crypts, and appeared almost totally dependent on the actions of TLR4. In conclusion, we present the *Sigirr^−/−^* mouse as an effective and exciting new model for the study of *C. jejuni* infection and pathogenesis. We speculate that our demonstration that *Sigirr^−/−^* mice can indeed be infected in a relevant fashion by *C. jejuni* will provide an impetus for further study, to better elucidate both the host factors and pathogenesis that drive gastroenteritis.

## Methods

### Bacterial strains and growing conditions

The wild-type *C. jejuni* strain used in this study is the commonly used 81–176 lab strain and all mutant and complemented strains were constructed on this background. The bacteria were routinely grown on Mueller-Hinton agar plates or broth, supplemented with the selective antibiotics Chloramphenicol and/or Kanamycin as required. Additionally, during mutant and complement construction, plates and broth were routinely supplemented with vancomycin (10 µg/mL) and trimethoprim (5 µg/mL) to prevent contamination. Cultures were routinely grown under microaerophilic conditions using anaerojars and CampyGen sachets (Oxoid) at 42°C.

### *C. jejuni* mutant and complement construction

To construct deletion mutants in the genes *flaA* and *kpsM*, each gene was PCR amplified with iProof (Bio-Rad) from *C. jejuni* 81–176 with the appropriate primers listed in Table S1. The product was polyA tailed and ligated to pGEM-T (Promega). Inverse PCR was performed on the resulting plasmid, deleting 1248 bp or 514 bp from the *flaA* or *kpsM* genes respectively. The *flaA* and *kpsM* inverse PCR products were digested with *Kpn*I and *Spe*I, or *Kpn*I and *Xba*I respectively, then ligated to the non-polar kanamycin resistance cassette (*aphA-3*) digested out of pUC18K-2 [57]. The construct was verified by sequencing and naturally transformed to *C. jejuni* 81–176. Mutant strains were selected by kanamycin resistance, and verified by sequencing.

To complement each of these mutants, *flaA* or *kpsM* was PCR amplified from *C. jejuni* 81–176 genomic DNA, digested with *Spe*I and *Mfe*I, or *Xba*I and *Mfe*I respectively, and inserted into pRRC [58] digested with *Xba*I and *Mfe*I. The resulting construct was verified by PCR and sequencing, and naturally transformed into the corresponding mutant. Complemented strains were selected on chloramphenicol and verified by PCR and sequencing.

Additional confirmation of the phenotypes of both mutant and complemented strains were undertaken to ensure they corresponded to previously published data for these mutants. The *ΔflaA* mutant and complement were tested for motility, indicating the mutant was only approximately 25% as motile as the WT or complemented strain [59]. The *ΔkpsM* mutant was tested for NaCl sensitivity [54] and hyper-biofilm formation [60], and the complement was confirmed to restore the wild-type phenotype for both. *In vitro* growth curves to confirm equal growth potential between both *ΔflaA* and *ΔkpsM* mutant and complemented strains were conducted in MH broth, at 37°C under microaerophilic conditions with samples taken at 6, 24, and 48 hours post inoculation.

### Mouse strains and infection experiments

The C57BL/6 (WT), *Sigirr^−/−^*, *Tlr2^−/−^*, *Tlr2^−/−^/Sigirr^−/−^*, *Tlr4^−/−^*, and *Tlr4^−/−^/Sigirr^−/−^* mouse strains used in this study were all bred in-house and kept under specific pathogen-free conditions at the Child and Family Research Institute (CFRI). The combined TLR and SIGIRR deficient mice were created by cross breeding single knockout strains as described previously [25]. Mice at 6–10 weeks of age were orally gavaged with 100 µl of a 50 mg/ml vancomycin solution suspended in PBS (dose per mouse of ∼5 mg). Four hours later, each mouse was inoculated with an overnight culture of ∼10^7^ CFUs of *C. jejuni* 81–176 or one of the above mentioned mutant strains. The weight of each mouse was recorded before antibiotic treatment and inoculation, and each mouse was weighed again every two days to check for weight loss/gain. Fecal samples were collected 1, 3, 5 and 7 DPI, were weighed, homogenized, serially diluted and plated onto *Campylobacter* agar plates containing Karmali selective supplements (Oxoid). Three and seven days post infection, mice were anaesthetized with isofluorane and euthanized by cervical dislocation. The mice were immediately dissected and their ileum, cecum, colon, mesenteric lymph nodes and spleen were isolated. Cecal and proximal colonic tissues were fixed in 10% neutral buffered formalin (Fisher). Cecal tissues were also washed to remove luminal contents and then suspended in RNAlater (Qiagen) for subsequent RNA extraction. The remainder of the cecum (including luminal contents), and other isolated tissue sections were suspended in 1 ml sterile PBS (pH 7.4) for viable cell counts. Tissue samples were homogenized, serially diluted and plated onto *Campylobacter* agar plates containing Karmali selective supplements (Oxoid). Following 48 hours incubation, at 42°C under microaerobic conditions colonies were enumerated, and the pathogen burden (CFUs/g of tissue) was calculated. Statistically significant differences were determined using a non-parametric Mann-Whitney test, with a p value below 0.05 used as the threshold for significance.

To monitor colonization of *C. jejuni* over a 25 day timeframe, three experimental groups comprising 13 WT and 15 *Sigirr^−/−^* mice total were inoculated with *C. jejuni* 81–176. Weights and fecal samples were taken every two days from 1 DPI to 25 DPI. CFUs present within the fecal samples were enumerated as described above and statistical significance was determined using multiple t-tests (p<0.05).

### Ethics statement

All animal experiments were performed according to protocol number A11-290, approved by the University of British Columbia's Animal Care Committee and in direct accordance with the Canadian Council of Animal Care (CCAC) guidelines. Mice were monitored for mortality and morbidity throughout their infection and euthanized if they showed signs of extreme distress or more than 15% body weight loss.

### Histology, pathological scoring and immunofluorescent staining

Tissues previously fixed in 10% formalin were paraffin embedded and cut for further histological analysis. The paraffin embedded tissue sections were stained with haematoxylin and eosin, and then photographed, and then used for pathological scoring. The scoring was done by two blinded observers according to previously established criteria [25]. Each tissue section was assessed for: (1) submucosal edema (0-no change, 1- mild, 2- moderate, 3- severe), (2) crypt hyperplasia (0-no change, 1: 1–50%, 2: 51–100%, 3: >100%), (3) goblet cell depletion (0-no change, 1-mild depletion, 2-severe depletion, 3-absence of goblet cells), (4) epithelial integrity (0-no pathological changes detectable, 1-epithelial desquamation (few cells sloughed, surface rippled, 2-erosion of epithelial surface (epithelial surface rippled, damaged), 3-epithelial surface severely disrupted/damaged, large amounts of cell sloughing, 4-ulceration (with an additional score of 1 added for each 25% fraction of tissue in the cross-section affected up to a maximum score of 8 (4+4) for a tissue section that had entirely lost its crypt structure due to epithelial cell loss and immune cell infiltration, (5) mucosal mononuclear cell infiltration (per 400× magnification field) (0-no change, 1- <20, 2- 20 to 50, 3- >50 cells/field), (6) submucosal PMN and mononuclear cell infiltration (per 400× magnification field) (1- <5, 2- 21 to 60, 3- 61 to 100, 4- >100 cells/field). A maximum score under this scale is 24. Statistical significance (p<0.05) was determined using a two-way ANOVA, with a Bonferroni post-test.

The paraffin embedded, formalin-fixed tissue sections were also used for immunofluorescent staining using variations on established protocols [25], [61]. Briefly, tissue sections were deparaffinized by heating for 8 minutes, clearing with xylene, rehydrating with 100%, 95%, and 70% ethanol, followed by dH_2_O. Antigen retrieval of the tissue sections was conducted with sodium citrate buffer (pH 6.0), in a steam bath for 30 minutes. Blocking was done with an endogenous Biotin-blocking kit (Molecular Probes) following manufacturer protocols, followed by 1 hour blocking with donkey serum blocking buffer (donkey serum in PBS containing 1% bovine serum albumin (BSA), 0.1% Triton-X100, 0.05% Tween 20, and 0.05% sodium azide). The primary antibodies used were for Actin (goat polyclonal, Santa Cruz Biotechnology), Cytokeratin 19 (goat polyclonal, Santa Cruz Biotechnology), and *Campylobacter jejuni* (Biotin-rabbit polyclonal, Abcam). Each was visualized using Alexa Fluor 488-conjugated donkey anti-goat IgG (Invitrogen) or Alexa Fluor 568-conjugated streptavidin (Molecular Probes). The tissues were mounted using ProLong Gold antifade reagent containing DAPI (Invitrogen). The stained slides were viewed using a Zeiss AxioImager Z1, photographed using an AxioCam HRm camera with AxioVision software. Confocal imaging was conducted with a Leica TCS SP5 system, using the Leica Application suite software.

Slides stained for *C. jejuni* and DAPI were used to assess crypt colonization. We used slides of formalin fixed, 7 day infected cecal tissues from WT, *Sigirr^−/−^*, *Tlr2^−/−^/Sigirr^−/−^*, and *Tlr4^−/−^/Sigirr^−/−^* mice to count the number of crypts containing visible numbers of *C. jejuni*. In total, 264, 146, 140, and 120 crypts were counted for each mouse strain respectively, from three slides each, each of which contained at least three tissue sections.

### RNA extraction and quantitative real-time PCR

Tissue samples previously isolated from infected or control mice were preserved in RNAlater at −20°C for later use. RNA was extracted using a Qiagen RNeasy kit (Qiagen) according to the manufacturer's protocol. The final RNA samples were eluted from the columns in sterile, RNAse free dH_2_O and quantified using an ND-1000 spectrophotometer (Nanodrop). cDNA was synthesized from the RNA using an Omniscripts RT kit (Qiagen) and Oligo-dT (Applied Biological Material Inc.). Quantitative real-time PCR was carried out using an MJ mini-opticon Real-Time PCR system (Bio-Rad) using IQ SYBR Green Supermix (Bio-Rad). The primers used have been described previously [25] and are listed in Table S1. Quantification of the qPCR results was performed using Gene Ex Macro OM 3.0 software (Bio-Rad) and ANOVAs were used to determined statistical significance of the results.

### HEK TLR reporter cell assays

HEK TLR reporter cell lines, HEK-Blue hTLR2 and HEK-Blue hTLR4, were purchased from InvivoGen (San Diego, CA, USA). HEK-Blue hTLR2 were obtained by co-transfection of hTLR2 and hCD14 co-receptor genes into HEK 293 cells, while HEK-Blue hTLR4 were obtained by co-transfection of hTLR4 and hMD-2/CD14 co-receptor genes. The cells were transfected with the secreted embryonic alkaline phosphatase (SEAP) gene and stably express SEAP under the control of a promoter inducible by NF-κB and activator protein 1 (AP-1). Thus, stimulation of hTLR2 or hTLR4 will lead to the production of extracellular SEAP in the culture medium proportional to the level of NF-κB/AP-1 activation. Cells were grown in High Glucose DMEM (HyClone, Logan, UT, USA) with 2 mM L-glutamine, 10% heat-inactivated FBS (HyClone), 100 µg/ml Normocin (InvivoGen) and selective antibiotics (1×HEK-Blue selection, InvivoGen) according to the manufacturer's instructions.

The activation of TLR2 or TLR4 was assessed by measuring the SEAP activity using QUANTI-Blue (InvivoGen) colorimetric assay. The reporter cells (5×10^4^/well) were seeded in a 96-well plate (BD Bioscience, Mississauga, ON, Canada). The next day, cells were treated with fresh media (without selective antibiotics) containing wild type, *ΔkpsM*, or *ΔkpsM+kpsM C. jejuni* strains for 4 h. Cells treated with culture medium only, TLR2 ligand Pam3CSK4 (100 ng/mL, InvivoGen) and TLR4 ligand lipopolysaccharide (LPS, Escherichia coli K-12, 100 ng/mL, InvivoGen) serve as the negative and positive controls, respectively. For each experiment, all conditions were done in triplicate. After 4 h incubation, culture media were collected and centrifuged to remove bacteria. The supernatants (20 µl) were then incubated with QUANTI-Blue solution (180 µl) in a 96-well flat-bottom plate at 37°C for 16–18 h to allow the color development. The color change of the substrate solution corresponds to the activation of NF-κB/AP-1, which can be quantified by optical density (λ = 655 nm) measurement using a SpectraMax 384 Plus plate reader (Molecular Devices, Sunnyvale, CA, USA).

## Supporting Information

Figure S1 **Colonization of WT and** ***Sigirr−/−*** **mouse ileum, colon and systemic sites by** ***C. jejuni*** **81–176, 3 and 7 DPI.** CFUs/g of *C. jejuni* 81–176 recovered from the ileum (A), colon (B), Mesenteric lymph nodes (MLN) (C), Spleen (D, fecal samples (E and F) and of infected mice. Relatively low and inconsistent numbers were recovered from the ileum of infected mice, while numbers comparable to the cecum were recovered from the colons. No significant difference was detected between WT and *Sigirr^−/−^* mice. n = 10 or 11 WT mice, and 12 or 13 *Sigirr^−/−^* mice for 3 and 7 DPI respectively.
(TIF)

Figure S2 **Relative bacterial composition, before and after vancomycin treatment.** Bacterial composition of mouse fecal samples taken before and after vancomycin treatment, and uninfected and infected with *C. jejuni* 81–176, as measured by qRT-PCR. Primers were specific to the major phyla *Bacteroidetes* and *Firmicutes*, as well as all *Eubacteria*, and the % of *Bacteroidetes* or *Firmicutes* was calculated relative to the total Eubacteria for each sample. The ratio of *Bacteroidetes* to *Firmicutes* shifted dramatically following vancomycin treatment (>40% to <1%), with the *Bacteroidetes* being reduced from the dominant phylum, to barely measurable quantities. However, no significant differences were noted between WT and *Sigirr^−/−^* mice under uninfected conditions or following infection with *C. jejuni* 81–176.
(TIF)

Figure S3 **Four week** ***C. jejuni*** **infection of WT and** ***Sigirr−/−*** **mice.** (A) CFUs of *C. jejuni* 81–176 recovered from the fecal samples of WT and *Sigirr^−/−^* mice over a period of 25 days, with fecal sampling taking place every two days from 1 DPI to 25 DPI. The experiment was repeated 3 times, for a total of 13 WT mice and 15 *Sigirr^−/−^* mice. The data displayed here is a representative experiment of the three, showing data from 4 WT and 8 *Sigirr^−/−^* mice. Slight differences between the clearance time between experiments led to higher variability at later time points between experiments, but results were consistent within each experiment. Colonization peaks at 7–9 DPI, then CFUs recovered from all the *Sigirr^−/−^* mice rapidly decline, with roughly half the *Sigirr^−/−^* mice clearing the infection by 23 DPI. WT mice did not exhibit any drop in pathogen burden by 25 DPI. A statistically significant difference between CFUs recovered from WT and *Sigirr^−/−^* mice (p<0.05) was measured between 13 and 25 DPI as determined by multiple t-tests. (B) The % change in mouse weight relative to their weight pre-inoculum over 25 days. No significant difference was found between WT and *Sigirr^−/−^* mice (p>0.05). n = 13 WT, 15 *Sigirr^−/−^* mice.
(TIF)

Figure S4 **Macroscopic images of mouse intestines infected by** ***C. jejuni*** **.** Images of the ceca and colons of mice colonized by *C. jejuni* 81–176, 3 DPI. The WT mouse did not show any outward signs of inflammation and was largely indistinguishable from that of an uninfected mouse. The infected *Sigirr−/−* mice show an enlargement of the mesenteric lymph nodes adjacent to the cecum and signs of inflammation around the cecum and proximal colon, but no signs of infection into the distal colon, or ileum. The *Tlr2^−/−^/Sigirr^−/−^* mice show a shrinkage of the cecum and colon, with no luminal content apparent. The mesenteric lymph nodes are enlarged, but the ileum still shows no outward signs of inflammation. The *Tlr4^−/−^/Sigirr^−/−^* mice show few signs of inflammation, but the cecum is often slightly enlarged, with more fluid contents.
(TIF)

Figure S5 **Mutant** ***C. jejuni*** **growth and colonization.** (A) Colonization of WT, *Tlr2^−/−^/Sigirr^−/−^*, and *Tlr4^−/−^/Sigirr^−/−^* by *C. jejuni ΔkpsM*. Results show that the pathogen burden of this mutant peaks at 7 DPI. Results are not substantially different than those observed in infected *Sigirr^−/−^* mice. n = 4 per condition. (B) *In vitro* growth by WT *C. jejuni* 81–176, *ΔflaA*, *ΔflaA+flaA*, *ΔkpsM* and *ΔkpsM+kpsM* in MH broth. No difference in growth was observed at 6, 24, or 48 hours growth as determined by multiple t-tests, p>0.05.
(TIF)

Figure S6 **Histology of TLR single knockouts.** H&E staining of formalin-fixed, paraffin-embedded cecal tissues of *Tlr2^−/−^* and *Tlr4^−/−^* mice 3 and 7 DPI. Neither mouse strain developed significant pathology 3 DPI, despite colonization comparable to their SIGIRR double knockout counterpart. At 7 DPI, the *Tlr4^−/−^* mice continued to show no pathology, while the *Tlr2^−/−^* mice did start to show signs of pathology, similar to their *Tlr2^−/−^/Sigirr^−/−^* counterpart.
(TIF)

Figure S7 **Cytokine expression in mice infected with** ***C. jejuni ΔkpsM.*** (A–H) qRT-PCR conducted on RNA extracted from the ceca of control or mice infected with *C. jejuni ΔkpsM*. Controls are the pooled results of 3, vancomycin pre-treated, but uninfected mice, euthanized 3 days post-treatment. All infected mice represent the average results of 2 independent experiments, each of which include the pooled RNA of 3–5 mice, euthanized either 3 or 7 DPI. Few mice exhibited elevated cytokine expression 3 DPI, however, expression was significantly higher in *Sigirr^−/−^* and *Tlr2^−/−^/Sigirr^−/−^* mice 7 DPI. Statistical significance was determined using a One way ANOVA with a Bonferroni post-test. * p<0.05 relative to WT (B6) or *Sigirr^−/−^* uninfected control mice. ** p<0.05 relative to the infected WT (B6) mice euthanized on the same DPI in addition to the uninfected control mice.
(TIF)

Table S1 **Primers used in this study were developed for this study or derived from previously published studies.** 16S rRNA primers designed by ^1^ Primer developed by Layton et al. 2006 [62], ^2^ Guo et al. 2008 [63], and ^3^ Fierer et al. 2005 [64].
(DOC)
