# Supplementary figures and images for: A Novel Mouse Model of Campylobacter jejuni Gastroenteritis Reveals Key Pro-inflammatory and Tissue Protective Roles for Toll-like Receptor Signaling during Infection
Source: PLoS Pathog. 2014 Jul 17;10(7):e1004264. doi: 10.1371/journal.ppat.1004264 (PMC4102570; doi:10.1371/journal.ppat.1004264)

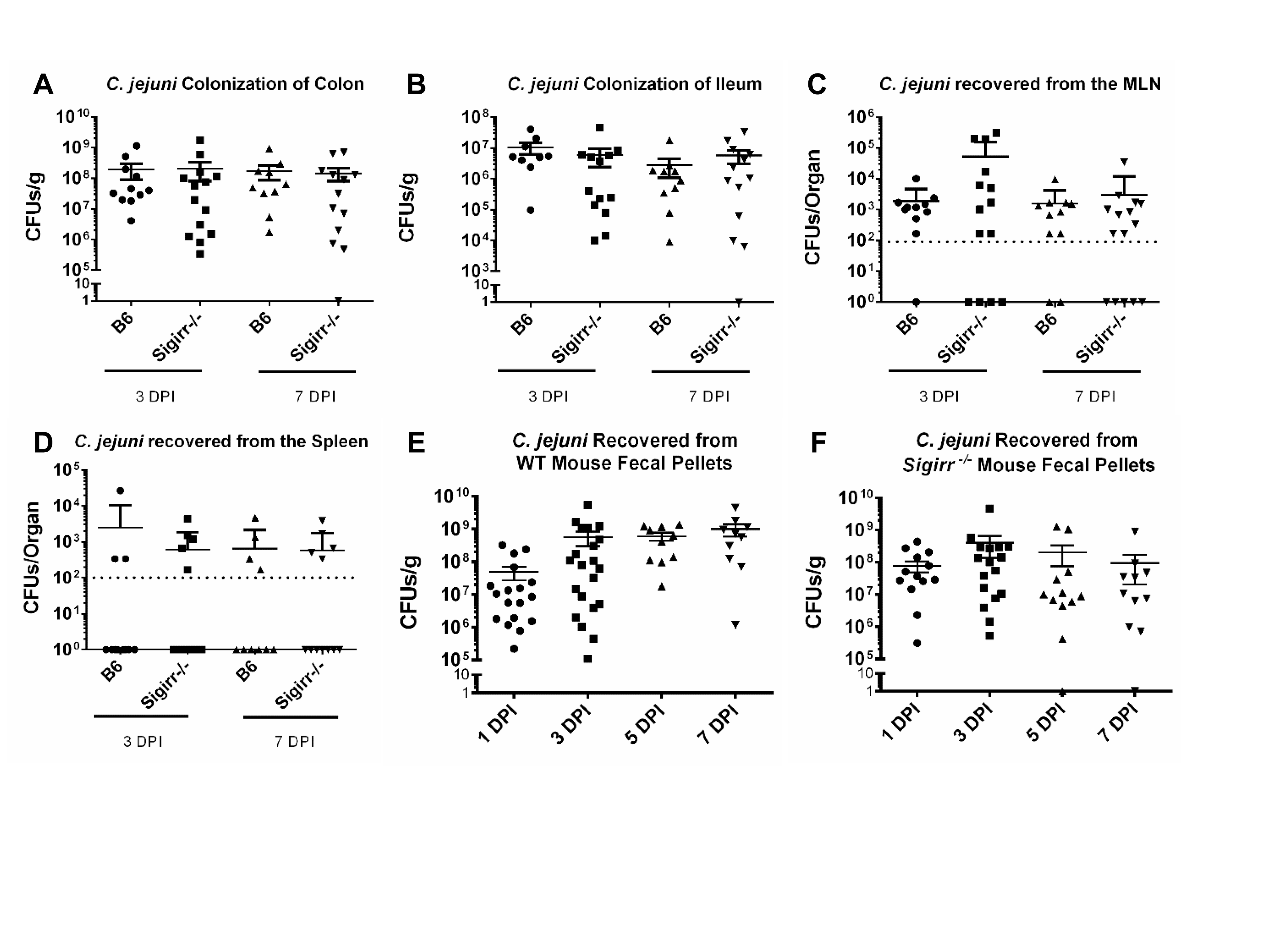

Supplement: Figure S1 — Colonization of WT and Sigirr−/− mouse ileum, colon and systemic sites by C. jejuni 81–176, 3 and 7 DPI. CFUs/g of C. jejuni 81–176 recovered from the ileum (A), colon (B), Mesenteric lymph nodes (MLN) (C), Spleen (D, fecal samples (E and F) and of infected mice. Relatively low and inconsistent numbers were recovered from the ileum of infected mice, while numbers comparable to the cecum were recovered from the colons. No significant difference was detected between WT and Sigirr−/− mice. n = 10 or 11 WT mice, and 12 or 13 Sigirr−/− mice for 3 and 7 DPI respectively. (TIF) [file ppat.1004264.s001.tif]

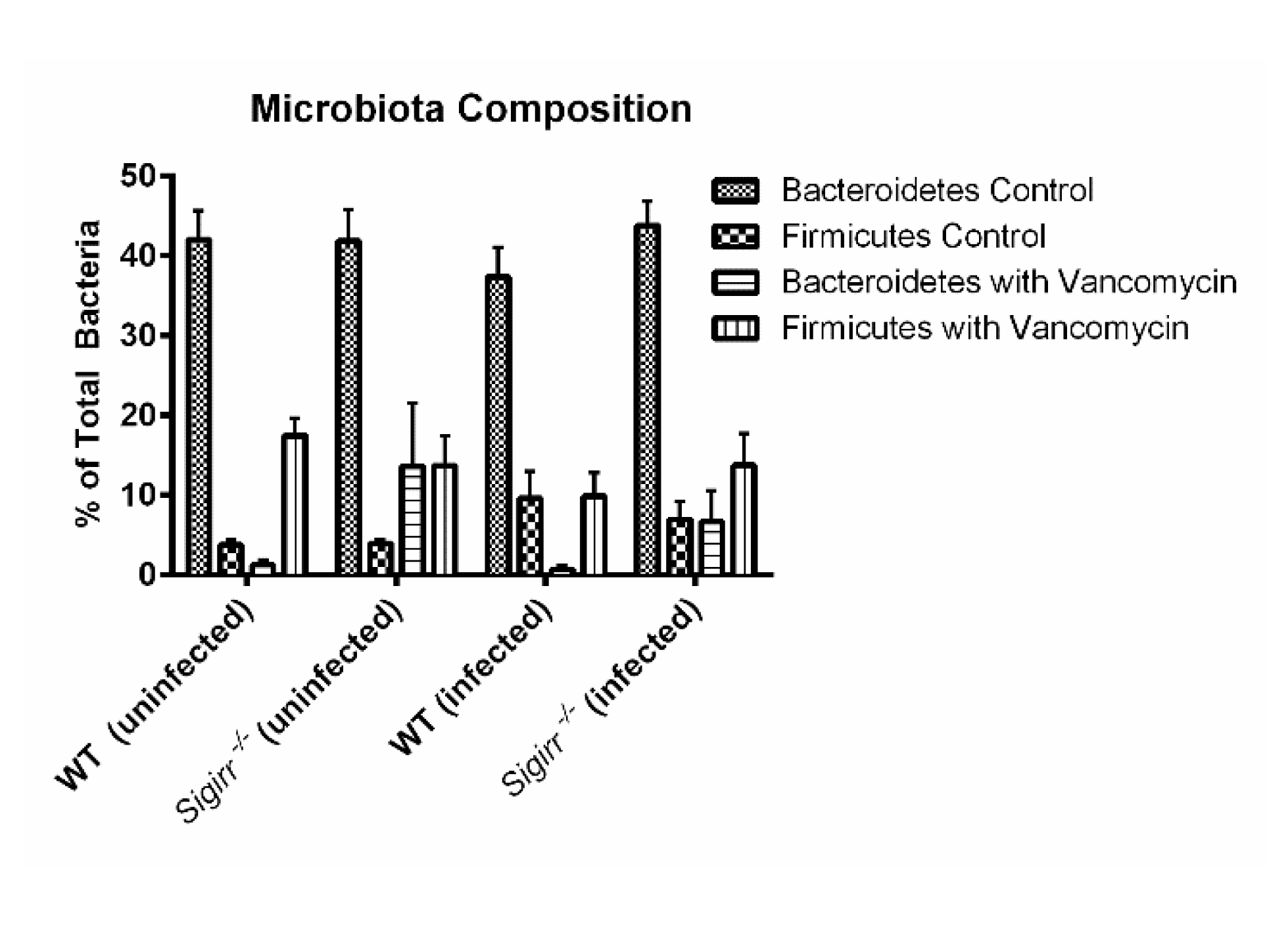

Supplement: Figure S2 — Relative bacterial composition, before and after vancomycin treatment. Bacterial composition of mouse fecal samples taken before and after vancomycin treatment, and uninfected and infected with C. jejuni 81–176, as measured by qRT-PCR. Primers were specific to the major phyla Bacteroidetes and Firmicutes, as well as all Eubacteria, and the % of Bacteroidetes or Firmicutes was calculated relative to the total Eubacteria for each sample. The ratio of Bacteroidetes to Firmicutes shifted dramatically following vancomycin treatment (>40% to <1%), with the Bacteroidetes being reduced from the dominant phylum, to barely measurable quantities. However, no significant differences were noted between WT and Sigirr−/− mice under uninfected conditions or following infection with C. jejuni 81–176. (TIF) [file ppat.1004264.s002.tif]

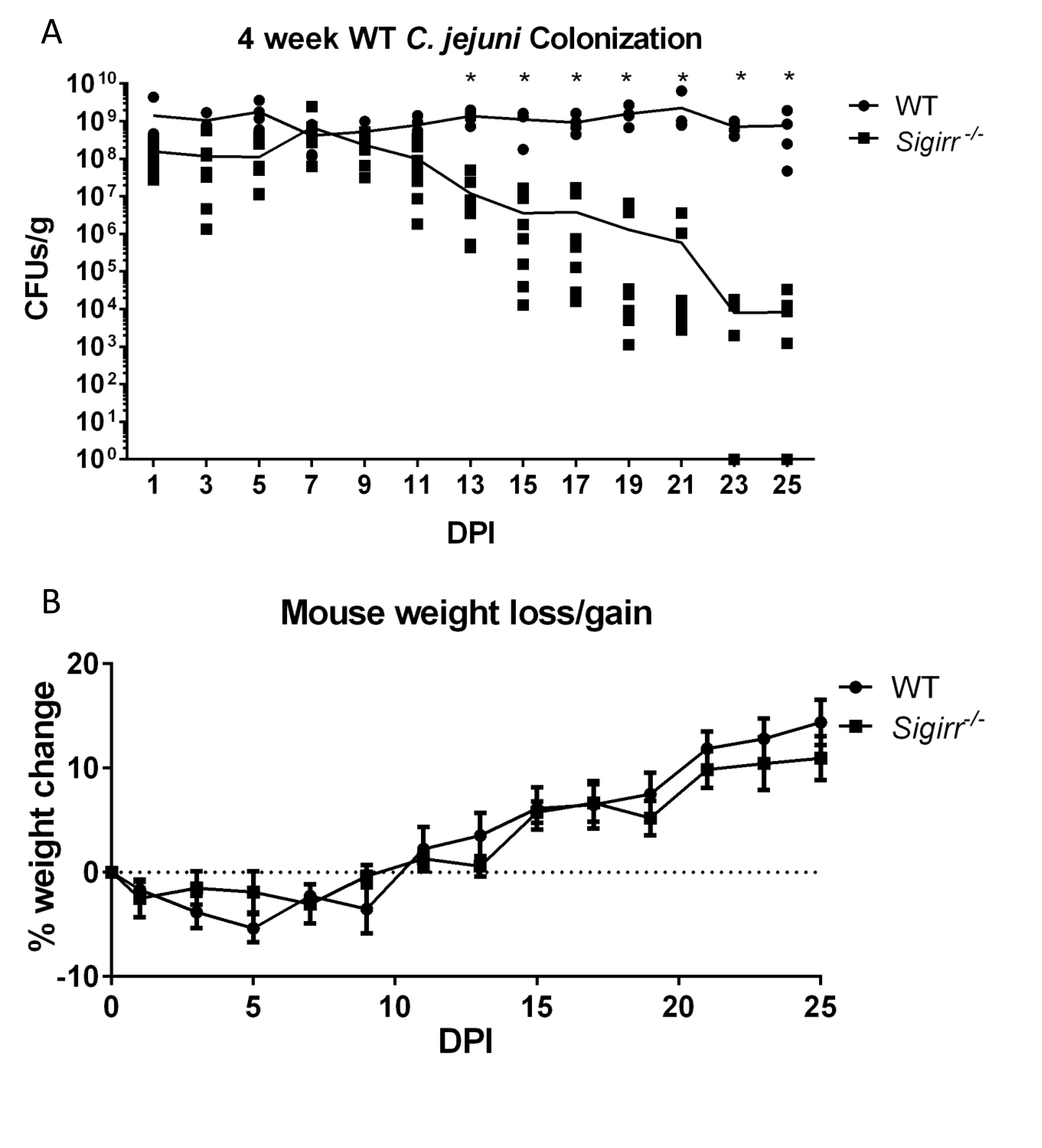

Supplement: Figure S3 — Four week C. jejuni infection of WT and Sigirr−/− mice. (A) CFUs of C. jejuni 81–176 recovered from the fecal samples of WT and Sigirr−/− mice over a period of 25 days, with fecal sampling taking place every two days from 1 DPI to 25 DPI. The experiment was repeated 3 times, for a total of 13 WT mice and 15 Sigirr−/− mice. The data displayed here is a representative experiment of the three, showing data from 4 WT and 8 Sigirr−/− mice. Slight differences between the clearance time between experiments led to higher variability at later time points between experiments, but results were consistent within each experiment. Colonization peaks at 7–9 DPI, then CFUs recovered from all the Sigirr−/− mice rapidly decline, with roughly half the Sigirr−/− mice clearing the infection by 23 DPI. WT mice did not exhibit any drop in pathogen burden by 25 DPI. A statistically significant difference between CFUs recovered from WT and Sigirr−/− mice (p<0.05) was measured between 13 and 25 DPI as determined by multiple t-tests. (B) The % change in mouse weight relative to their weight pre-inoculum over 25 days. No significant difference was found between WT and Sigirr−/− mice (p>0.05). n = 13 WT, 15 Sigirr−/− mice. (TIF) [file ppat.1004264.s003.tif]

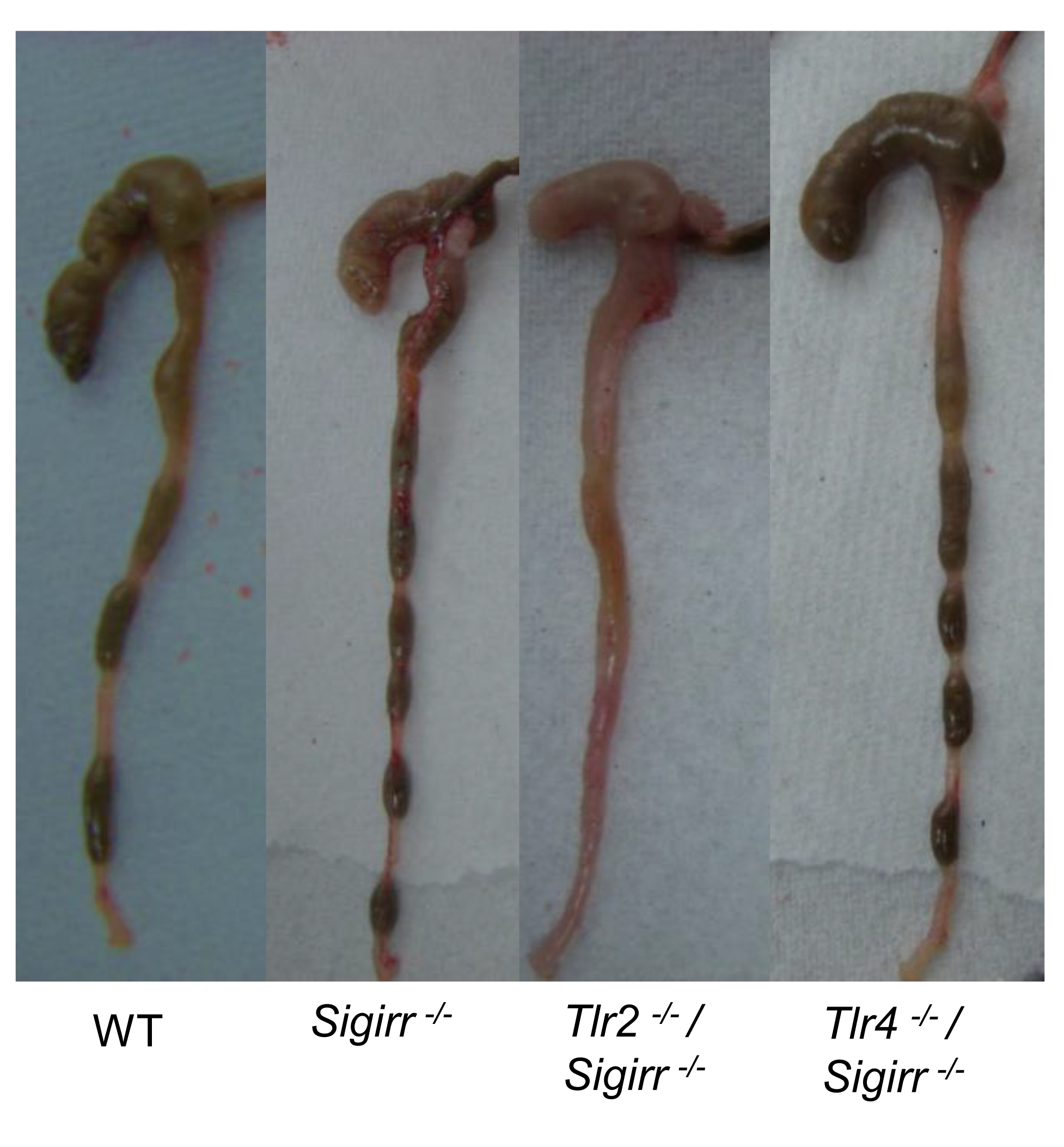

Supplement: Figure S4 — Macroscopic images of mouse intestines infected by C. jejuni . Images of the ceca and colons of mice colonized by C. jejuni 81–176, 3 DPI. The WT mouse did not show any outward signs of inflammation and was largely indistinguishable from that of an uninfected mouse. The infected Sigirr−/− mice show an enlargement of the mesenteric lymph nodes adjacent to the cecum and signs of inflammation around the cecum and proximal colon, but no signs of infection into the distal colon, or ileum. The Tlr2−/−/Sigirr−/− mice show a shrinkage of the cecum and colon, with no luminal content apparent. The mesenteric lymph nodes are enlarged, but the ileum still shows no outward signs of inflammation. The Tlr4−/−/Sigirr−/− mice show few signs of inflammation, but the cecum is often slightly enlarged, with more fluid contents. (TIF) [file ppat.1004264.s004.tif]

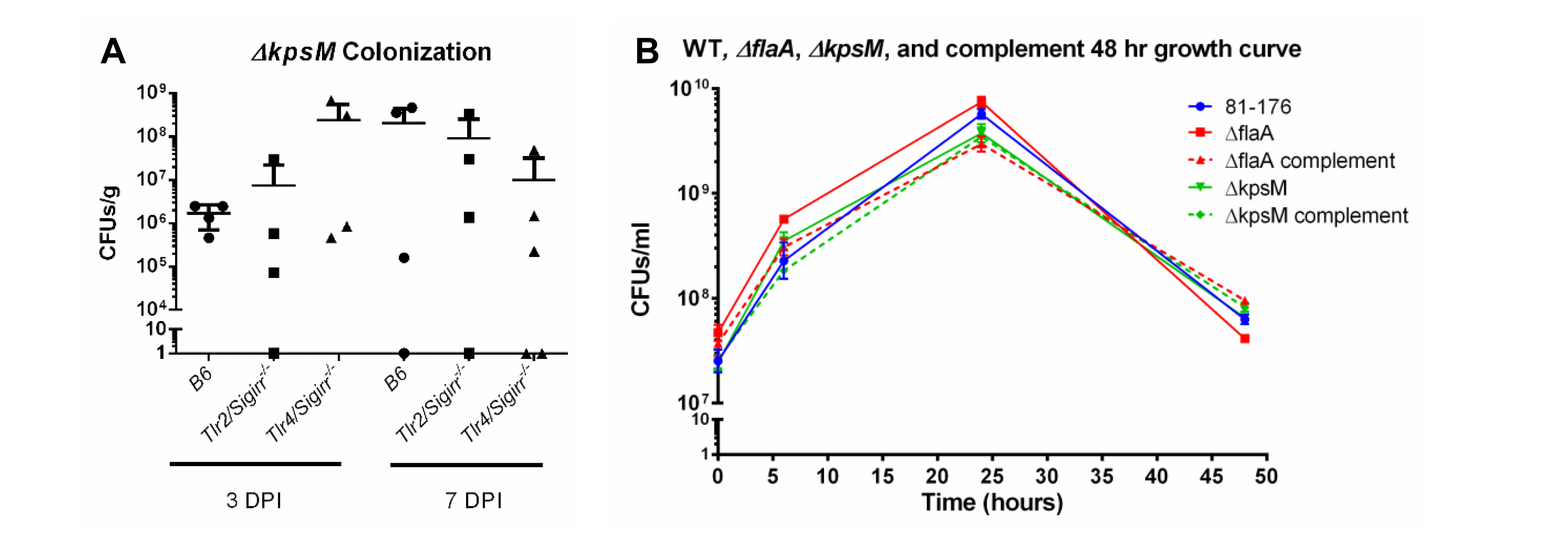

Supplement: Figure S5 — Mutant C. jejuni growth and colonization. (A) Colonization of WT, Tlr2−/−/Sigirr−/−, and Tlr4−/−/Sigirr−/− by C. jejuni ΔkpsM. Results show that the pathogen burden of this mutant peaks at 7 DPI. Results are not substantially different than those observed in infected Sigirr−/− mice. n = 4 per condition. (B) In vitro growth by WT C. jejuni 81–176, ΔflaA, ΔflaA+flaA, ΔkpsM and ΔkpsM+kpsM in MH broth. No difference in growth was observed at 6, 24, or 48 hours growth as determined by multiple t-tests, p>0.05. (TIF) [file ppat.1004264.s005.tif]

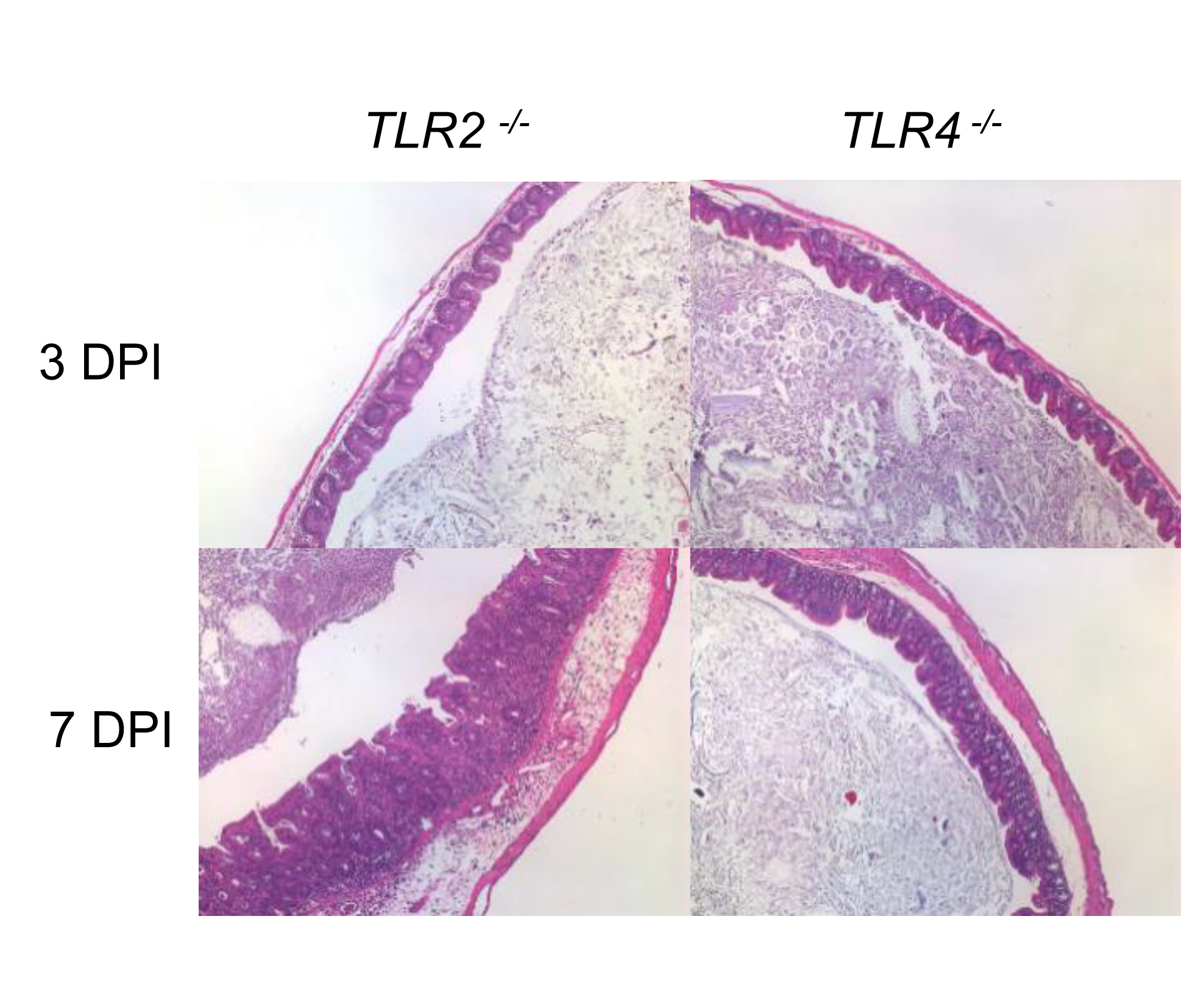

Supplement: Figure S6 — Histology of TLR single knockouts. H&E staining of formalin-fixed, paraffin-embedded cecal tissues of Tlr2−/− and Tlr4−/− mice 3 and 7 DPI. Neither mouse strain developed significant pathology 3 DPI, despite colonization comparable to their SIGIRR double knockout counterpart. At 7 DPI, the Tlr4−/− mice continued to show no pathology, while the Tlr2−/− mice did start to show signs of pathology, similar to their Tlr2−/−/Sigirr−/− counterpart. (TIF) [file ppat.1004264.s006.tif]

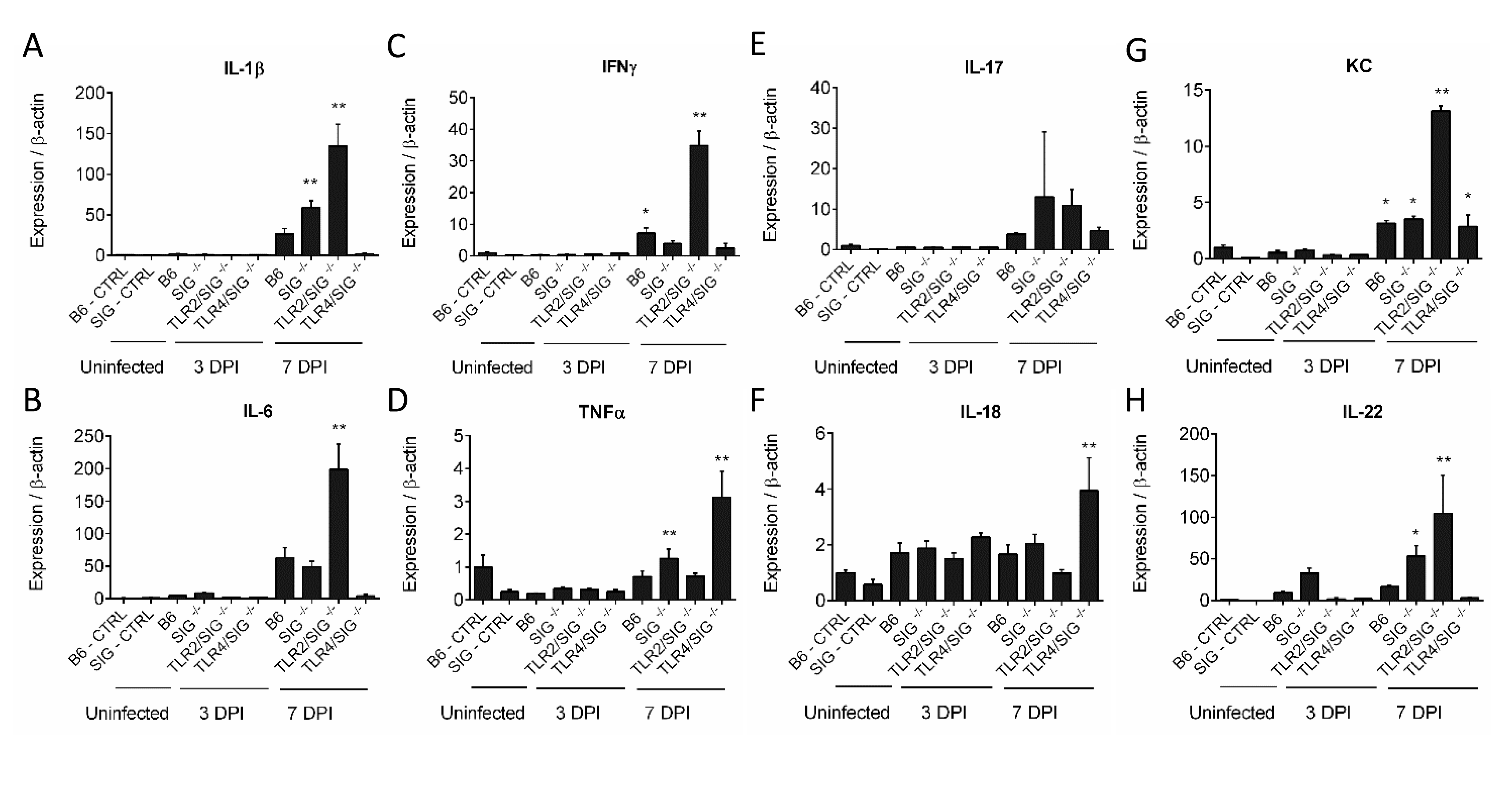

Supplement: Figure S7 — Cytokine expression in mice infected with C. jejuni ΔkpsM. (A–H) qRT-PCR conducted on RNA extracted from the ceca of control or mice infected with C. jejuni ΔkpsM. Controls are the pooled results of 3, vancomycin pre-treated, but uninfected mice, euthanized 3 days post-treatment. All infected mice represent the average results of 2 independent experiments, each of which include the pooled RNA of 3–5 mice, euthanized either 3 or 7 DPI. Few mice exhibited elevated cytokine expression 3 DPI, however, expression was significantly higher in Sigirr−/− and Tlr2−/−/Sigirr−/− mice 7 DPI. Statistical significance was determined using a One way ANOVA with a Bonferroni post-test. * p<0.05 relative to WT (B6) or Sigirr−/− uninfected control mice. ** p<0.05 relative to the infected WT (B6) mice euthanized on the same DPI in addition to the uninfected control mice. (TIF) [file ppat.1004264.s007.tif]
