# Supplementary material for: A Novel Mouse Model of Campylobacter jejuni Gastroenteritis Reveals Key Pro-inflammatory and Tissue Protective Roles for Toll-like Receptor Signaling during Infection
Source: PLoS Pathog. 2014 Jul 17;10(7):e1004264. doi: 10.1371/journal.ppat.1004264 (PMC4102570; doi:10.1371/journal.ppat.1004264)
Supplement: Table S1 — Primers used in this study were developed for this study or derived from previously published studies. 16S rRNA primers designed by 1 Primer developed by Layton et al. 2006 [62], 2 Guo et al. 2008 [63], and 3 Fierer et al. 2005 [64]. (DOC) [file ppat.1004264.s008.doc]

| **Supplemental Table 1: Primer List** | | | |
| --- | --- | --- | --- |
| **Primer Name** | **Sequence ( 5’-3’)** | **Restriction enzyme** | |
| **Mutant Construction** | | | |
| flaA-L1 | AAA CTT TAA CAA CCA ATC GTG G | |  |
| flaA-R2 | AAG CAA GAA GTG TTC CAA GTT | |  |
| flaA-IL | acg **ggt acc** ACC TGA ACT AAG TCT GCT TAA AG | | KpnI |
| flaA-IR2 | gcg **act agt** TTC AGG ATT TTC TTC TGG CTC | | SpeI |
| flaA-L4 | ata **caa ttg** GTT AGT AAA ATT GAA GAT GAA AGA GAG T | | MfeI |
| flaA-R4 | gcg **act agt** AAG CAA GAA GTG TTC CAA GTT | | SpeI |
| kpsM-L5 | AAA GGT GTT TAT ACG GCT AGT G | |  |
| kpsM-R5 | TAG CAC TCA TTC CCG AAG AA | |  |
| kpsM-IL | acg **ggt acc** AAA AGC ACA ACG CTC ATA GG | | KpnI |
| kpsM-IR | gcg **tct aga** GGG CTT TTT ATT TAC TAC TAT AAT AGA CA | | XbaI |
| kpsM-CODE | acac **tct aga** CAA TGC TTT AGG ACT TAG TAA AAA GC | | XbaI |
| kpsT-COMP | acac **caa ttg** TTA CAT ATC TTG GTG CTG CAA TC | | MfeI |
| **qPCR-Primers** | | | |
| AllBac296F^1^ | GAG AGG AAG GTC CCC CAC | | |
| AllBac412R^1^ | CGC TAC TTG GCT GGT TCA G | | |
| Firm934F1^2^ | GGA GYA TGT GGT TTA ATT CGA AGC A | | |
| Firm1060R^2^ | AGC TGA CGA CAA CCA TGC AC | | |
| UniF340^3^ | ACT CCT ACG GGA GGC AGC AGT | | |
| UniR514^3^ | ATT ACC GCG GCT GGC | | |
| IL-1BF | CAG GAT GAG GAC ATG AGC ACC | | |
| IL-1BR | CTC TGC AGA CTC AAA CTC CAC | | |
| IL-6F | GAG GAT ACC ACT CCC AAC AGA CC | | |
| IL-6R | AAG TGC ACT ACT GTT GTT CAT ACA | | |
| IFN-gF | TCA AGT GGC ATA GAT GTG GAA GAA | | |
| IFN-gR | TGG CTC TGC AGG ATT TTC ATG | | |
| TNF-aF | CAT CTT CTC AAA ATT CGA GTG ACA A | | |
| TNF-aR | TGG GAG TAG ACA AGG TAC AAC CC | | |
| IL-17AF | GCT CCA GAA GGC CCT CAG A | | |
| IL-17AR | CTT TCC CTC CGC ATT GAC A | | |
| IL-18F | GCC ATG TCA GAA GAC TCT TGC |  | |
| IL-18R | GTA CAG TGA AGT CGG CCA AAG TTG T |  | |
| KCF | TGC ACC CAA ACC GAA GTC AT |  | |
| KCR | TTG TCA GAA GCC AGC GTT CAC |  | |
| IL-22F | ACC TTT CCT GAC CAA ACT CA |  | |
| IL-22R | AGC TTC TTC TCG CTC AGA CG |  | |

^1^ Layton et al. 2006. ^2^ Guo et al. 2008. ^3^ Fierer et al. 2005.
